# Professionals’ views on the justification for esophageal adenocarcinoma screening: A systematic literature search and qualitative analysis

**DOI:** 10.1016/j.pmedr.2023.102264

**Published:** 2023-05-26

**Authors:** Jasmijn Sijben, Yonne Peters, Linda Rainey, Mejdan Gashi, Mireille J.M. Broeders, Peter D. Siersema

**Affiliations:** a Department of Gastroenterology and Hepatology, Radboud University Medical Center, Nijmegen, The Netherlands; b Department for Health Evidence, Radboud University Medical Center, Nijmegen, The Netherlands; c Dutch Expert Center for Screening, Nijmegen, The Netherlands; d Department of Gastroenterology and Hepatology, Erasmus MC - University Medical Center, Rotterdam, The Netherlands

**Keywords:** Early detection of cancer, Esophageal neoplasms, Barrett esophagus, Health knowledge, attitudes, practice

## Abstract

Screening for early esophageal adenocarcinoma (EAC), including screening for its precursor Barrett’s esophagus (BE), has the potential to reduce EAC-related mortality and morbidity. This literature review aimed to explore professionals’ views on the justification for EAC screening. A systematic search of Ovid Medline, EMBASE, and PsycInfo, from January 1, 2000 to September 22, 2022, identified 5 original studies and 63 expert opinion articles reporting professionals’ perspectives on EAC screening. Included articles were qualitatively analyzed using the framework method, which was deductively led by modernized screening principles. The analyses showed that many professionals are optimistic about technological advancements in BE detection and treatment. However, views on whether the societal burden of EAC merits screening were contradictory. In addition, knowledge of the long-term benefits and risks of EAC screening is still considered insufficient. There is no consensus on who to screen, how often to screen, which screening test to use, and how to manage non-dysplastic BE. Professionals further point out the need to develop technology that facilitates automated test sample processing and public education strategies that avoid causing disproportionately high cancer worry and social stigma. In conclusion, modernized screening principles are currently insufficiently fulfilled to justify widespread screening for EAC. Results from future clinical screening trials and risk prediction modeling studies may shift professionals’ thoughts regarding justification for EAC screening.

## 1 Introduction

Since the 1990s, an increasing incidence of esophageal adenocarcinoma (EAC) has been observed in Western countries. The overall 5-year survival rate remains less than 20% due to detection at an advanced stage (Peters et al., 2019, GBD Oesophageal Cancer Collaborators, 2020). For this reason, it has been suggested to screen high-risk individuals for the presence of Barrett’s esophagus (BE), which is the precursor of EAC (Peters et al., 2019). Those with BE can be followed up to detect incident dysplasia and early EAC (Fitzgerald et al., 2014, Shaheen et al., 2022, Qumseya et al., 2019). Subsequent endoscopic resection of early EAC or eradication of detected dysplasia may ultimately reduce the incidence, morbidity and mortality associated with EAC (Shaheen et al., 2009, Codipilly et al., 2018). High-definition upper endoscopy (EGD) combined with pathological assessment of biopsies is considered the gold standard for identification of BE, dysplasia, and EAC, although this test is considered too invasive and costly to screen large populations.

Recently, alternative screening tests more suitable to screen larger populations are being developed. First, transnasal endoscopy (TNE) is performed with an ultrathin endoscope inserted through the nose and performed without sedation. TNE can accurately diagnose BE, but there is no consensus on whether the small biopsies are sufficient to identify dysplasia (Huibertse et al., 2022). Second, the Cytosponge-TFF3, EsoCheck, and other non-endoscopic cell-collection devices comprise either an encapsulated sponge or a balloon attached to a thread (Ross-Innes et al., 2015, Moinova et al., 2018). Biomarker panels can be applied to on the collected cells to identify BE. Third, an ‘electronic nose’ device can be used to measure exhaled volatile organic compounds, byproducts of (patho)physiologic processes in cells, which has shown promising diagnostic accuracy for detecting BE (Peters et al., 2020).

Screening is more than applying a screening test. Assessment of the justification for screening policies is conventionally based on principles first described by Wilson and Jungner (World Health Organization, 1966). Previous studies that used these principles to evaluate EAC screening (performed in 2002 and 2005) concluded that endoscopic screening should not be endorsed, mainly due to lacking evidence that screening is beneficial and the lack of a well-characterized target population (Dellon and Shaheen, 2005, Shaheen et al., 2002).

Views on the justification for screening may vary over time and change with new evidence. For example, the increasing incidence of EAC may have changed professionals’ views on the burden of the disease – and thus the perceived likelihood that screening could be beneficial. Furthermore, the development of novel screening tests may have changed views on the performance of available test options (Fitzgerald et al., 2020). Table 1 illustrates that screening recommendations in guidelines are also dynamic (Fitzgerald et al., 2014, Shaheen et al., 2022, Playford, 2006, Wang and Sampliner, 2008, Shaheen et al., 2016, Spechler et al., 2011). The British Society of Gastroenterology (BSG) and the American College of Gastroenterology (ACG) have switched from not recommending screening in the 2000s to endorsing screening for a high-risk population in the past decade. The most recent ACG guideline is the first to endorse the application of non-endoscopic cell-collection devices for ‘once-in-a-lifetime’ examination of high-risk individuals (Shaheen et al., 2022). However, international guidelines are not consistent: the 2019 guideline by the American Society for Gastrointestinal Endoscopy (ASGE) and 2011 guideline by the American Gastroenterological Association (AGA) emphasize the lack of randomized clinical trials (RCTs) that support BE screening (Qumseya et al., 2019, Spechler et al., 2011).

**Table 1:** Former and current Gastroenterology and Endoscopy Society guidelines for BE screening.

**Society**	**Year**	**Screening Recommendation**	**Screening test**	**Screening population**
	*Former*			
British Society of Gastroenterology (Playford, 2006)	2006	Not recommended	Endoscopy	–
American College of Gastroenterology (Wang and Sampliner, 2008)	2008	Insufficient evidence	Endoscopy	–
American College of Gastroenterology (Shaheen et al., 2016)	2016	Consider	Endoscopy	Men > 5 y GERD, or with more than weekly symptoms + ≥2 risk factors: >50 y, central obesity, Caucasian, smoking, first-degree relative with BE or EAC
	*Current*			
British Society of Gastroenterology (Fitzgerald et al., 2014)	2014	Consider	Endoscopy	GERD with ≥ 3 risk factors: >50 y, Caucasian, male gender, obesity, family history
American Gastroenterological Association (Spechler et al., 2011)	2011	Insufficient evidence	Endoscopy	–
American Society for Gastrointestinal Endoscopy (Qumseya et al., 2019)	2019	Insufficient evidence. However, if performed, suggested to target an at-risk population.	Endoscopy	Individuals with a family history of EAC or BE (high risk) OR GERD with ≥ 1 risk factors (moderate risk): >50 y, male gender, Caucasian, smoking, obesity
American College of Gastroenterology (Shaheen et al., 2022)	2022	Single examination suggested	Endoscopy or non-endoscopic capsule sponge device combined with a biomarker	Chronic GERD with ≥ 3 risk factors: male sex, age > 50 y, White race, tobacco smoking, obesity, a first-degree relative with BE or EAC

GERD, gastro-esophageal reflux; BE, Barrett’s esophagus; EAC, esophageal adenocarcinoma.

Systematic re-evaluation of the fulfillment of screening principles will help guide complex decisions surrounding the implementation of novel screening tests and identify knowledge gaps. The objectives of this literature review were to systematically explore and analyze health care professionals’ views on the justification for EAC screening and how these views developed over time.

## 2 Review methods

### 2.1 Systematic literature search

Typical of a newly developing area, original research exploring professionals’ views on EAC screening is scarce. We, therefore, searched for both original studies and expert opinion articles, such as editorials, letters, and narrative reviews from which the author’s perspective on EAC screening could be extracted. Three electronic databases, Ovid Medline/PubMed, Ovid EMBASE and PsychInfo, were searched for the period January 1, 2000 – September 22, 2022. The search strategies were database-specific and developed in consultation with an experienced medical information specialist. We included a combination of subject headings and free-text terms for “Barrett’s esophagus”, “esophageal neoplasm or adenocarcinoma”, “mass screening”, “early detection of cancer”, “physician practice patterns”, “facilitator”, “barrier”, “implication”, “ethics”, “legislation and jurisprudence”, “cultural factor”, “social factor” and “qualitative research” in the title and abstract (Supplementary Table 1). The reference lists of all included studies were reviewed. Two researchers (JS and MG) independently screened each identified article for eligibility. An article was excluded if: 1) it was a systematic review or cost-effectiveness analysis, 2) it only reported perceptions of the public, or 3) it only addressed issues important for developing countries.

### 2.2 Definitions

EAC screening was defined as offering screen-eligible individuals a test aimed at detection of BE and/or BE-related neoplasia, with the intention to follow-up identified BE patients and treat dysplasia and early cancer once diagnosed. Screen-eligibility was deliberately not further specified to guarantee an encompassing overview.

The term *screening program* is inconsistently used. In this review, we adhere to the European Council’s definitions of screening program status (Ponti et al., 2017). EAC screening should currently be classified as *non-program screening*, defined as any examination for early detection of cancer performed in a clinical context. The screening policy requires some degree of public responsibility, organization, and supervision to receive a program status. It would therefore need to be publicly documented and funded, for example by the National Health Service (in the UK).

### 2.3 Data analysis

Data from original studies were summarized in an evidence table. We utilized the framework method to qualitatively analyze expert opinion articles, which entails a systematic method of selecting and organizing text based on key themes (Ritchie et al., 2013). The framework analysis was deductively led by a modernized version of Wilson and Junger’s principles that were previously identified through a systematic literature review and a Delphi consensus process (Dobrow et al., 2018). The following key themes are incorporated in the modernized set of screening principles: 1) epidemiology of the condition, 2) natural history of the condition, 3) target population, 4) screening test performance, 5) interpretation of test results, 6) post-screening test options, 7) infrastructure, 8) coordination and integration, 9) acceptability and ethics, 10) benefits and harms, 11) economic evaluation, and 12) quality. With these themes in mind, we extracted relevant text fragments from expert opinion articles. In parallel, the extracted text was inductively and iteratively coded to capture concepts relevant for EAC screening across included articles. The codes and accompanying text fragments, authors, and publication years were subsequently mapped back on the twelve screening principles to create the framework matrix (accessible via https://doi.org/10.17026/dans-x67-9bzx). Two independent researchers (JS and MG) performed each step, with discrepancies resolved through discussion. We managed qualitative data using ATLAS.ti version 8.4.20.

To illustrate if professionals’ views on EAC screening varied over time, we mapped their screening recommendations by calendar year. We used a two-stage process including extraction of the text fragment containing a recommendation from each article (independently, by JS and MG), followed by a classification of the recommendation in five predefined categories (independently and blinded to the author and publication year, by YP and PS). The following categories were applied: ++, recommending; +, motivation; +/−, neutral position; −, serious doubt; −/−, recommending against. We used SPSS (version 25; IBM Corporation, Armonk, NY) to calculate Cohen’s kappa values to assess inter-rater agreement.

### 2.4 Quality appraisal

The data relevant to this review were expected to be mainly drawn from narrative texts that reflected the author’s perspective on EAC screening. Thus, traditional quality assessment of included studies was not considered appropriate.

## 3 Results

### 3.1 Included articles

**Fig. 1:**
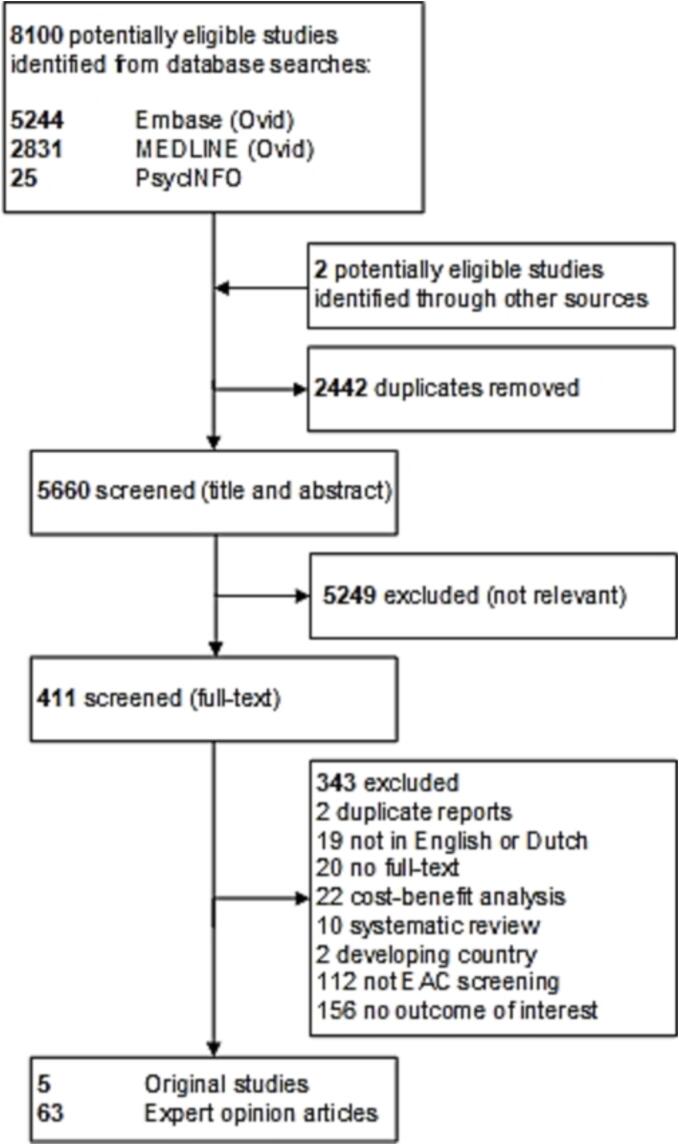
Study selection.

Of the 8100 references identified in the literature search, 411 were included after title and abstract assessment (by JS and MG, moderate inter-rater agreement κ = 0.51; SE = 0.026) (Fig. 1). After full-text assessment, 68 articles were selected (by JS and MG, substantial inter-rater agreement κ = 0.73; SE = 0.043); including 5 (7%) original studies and 63 (93%) expert opinion articles (Supplementary Table 2). All original studies were performed in the US (Table 2). Corresponding authors of expert opinion articles were from the US (68%), the UK (17%), other European countries (13%), and Australia (2%).

**Table 2:** Original studies reporting professionals’ views on BE/EAC screening.

**Reference**	**Country**	**Study design**	**Test**	**Professional types and sample size**	**Key findings relevant to this review**
Boolchand et al., 2006	US	Cross-sectional survey	EGD	PCPs (n = 271) Internists (n = 215)Other (n = 56)	• Internists were more likely than family practitioners to refer patients with GERD for endoscopic evaluation (26% vs. 16%, p = 0.005) • Reasons for EGD referral: risk factors, duration/refractoriness/frequency of GERD symptoms, alarm symptoms, diagnostic uncertainty • Reasons for EGD non-referral: cost, poor patient acceptance, insufficient evidence for BE screening, risk of complications • Interest to perform unsedated esophagoscopy in office: 52% of PCPs
Chey et al., 2005	US	Cross-sectional survey	EGD	PCPs (n = 1046)	• 87% agreed that patients with GERD (>5 y) should be screened for BE
Kolb et al., 2022	US	Cross-sectional survey	EGD	GIs (n = 120) PCPs (n = 195)	• BE screening is effective for early esophageal cancer detection: 72% of GIs, 71% of PCPs agreed • BE screening reduces all-cause mortality: 23% of GIs, 22% of PCPs agreed • BE screening is cost-effective for at-risk individuals: 56% of GIs, 38% of PCPs agreed • Not performing BE screening poses malpractice liability: 41% of GIs, 26% of PCPs agreed • PCPs should not order BE screening based on lack of recommendation from the USPSTF: 17% of GIs, 29% of PCPs agreed • Better data on the benefits of BE screening are needed: 75% of GIs, 66% of PCPs agreed • Better data on the harms of BE screening are needed: 67% of GIs, 59% of PCPs agreed • A randomized trial on BE screening would impact my decision to refer patients: 90% of GIs, 80% of PCPs agreed • BE screening has equally strong supporting data as CRC screening: 6% of GIs, 5% of PCPs agreed • Provider barriers: difficulty identifying at-risk patients, lack of knowledge of guidelines, ineffective treatment, not my responsibility, competing concerns, insufficient clinic time, unsure about insurance coverage, patient disinterest, patients don’t understand, patient non-adherence
Lin et al., 2002	US	Cross-sectional survey	EGD	GIs (n = 162)	• 87% screened patients with GERD (>1 y) • 72% believed this is efficacious • 48% believed this is cost-effective • Reasons for screening despite disbelieve in effectiveness: patient request, procedure reimbursement, medicolegal
Rubenstein et al., 2008	US	Cross-sectional survey	EGD	GIs (n = 224)	• Clinical practice: 98% would screen males (55 y) with GERD (20 y); 42% would screen females (55 y) with GERD (2 y) • Median perceived preventable deaths following screening and surveillance for EAC: 30% (IQR, 20–50%) • Median perceived preventable deaths following screening for CRC: 75% (IQR, 50–80%) • 85% believed EAC screening is less efficacious than CRC screening • Prior malpractice suit was associated with more aggressive screening and surveillance (OR; 3.6, 95% CI; 1.1–12)

Conventional upper endoscopy, EGD; PCP, primary care provider; GI, gastroenterologist; GERD, gastro-esophageal reflux; BE, Barrett’s esophagus; EAC, esophageal adenocarcinoma; IQR, interquartile range; CRC, colorectal cancer; OR, odds ratio.

### 3.2 Screening approach

A variety of potential EAC screening approaches were described in included articles:

#### 3.2.1 Targeted screening | clinician’s judgement vs. systematic approach

Targeted screening is only offered to individuals at increased risk for EAC, such as individuals with gastro-esophageal reflux disease (GERD) or other risk factors for BE/EAC. Several articles suggest that such high-risk individuals could undergo a screening test that is either self-initiated or initiated during a regular office visit, thus depending on *individual clinician’s judgment* of eligibility (Boolchand et al., 2006, Chey et al., 2005, Kolb et al., 2022, Lin et al., 2002, Rubenstein et al., 2008, Adams et al., 2014, Atkinson and Chak, 2010, Craanen and Kuipers, 2001, Crockett et al., 2010, Cross, 2011, Falk, 2019, Gerson, 2011). Some articles describe a scenario in which a health care organization *systematically* invites a group of at-risk individuals to undergo screening (di Pietro et al., 2018, Lao-Sirieix and Fitzgerald, 2012, O’Donovan and Fitzgerald, 2018, Vaughan and Fitzgerald, 2015, Yusuf and Fitzgerald, 2021).

#### 3.2.2 Population screening | combining with colorectal cancer screening

Population screening is offered to everyone in a predefined age group, regardless of other risk factors (Patel and Gyawali, 2019, Bretthauer and Kalager, 2013, Graham et al., 2016, Malagelada, 2011). A minority of professionals have suggested integrating EAC screening into established colorectal cancer population screening programs (Graham and Tan, 2020, Desai et al., 2021). One article suggested to combine this strategy with ablation of all Barrett’s mucosa along with tailored acid-suppressive–antireflux therapy to prevent recurrence (Graham and Tan, 2020).

### 3.3 Findings from original studies

Characteristics and findings from original studies are summarized in Table 2. All included original studies were surveys conducted in the US, addressed EAC screening with conventional upper endoscopy, and included clinicians (primary care providers [PCPs], gastroenterologists, or internists). In these surveys, 16%–98% of clinicians screened patients with GERD (Boolchand et al., 2006, Chey et al., 2005, Lin et al., 2002, Rubenstein et al., 2008), 71%–72% believed that screening for BE is effective for early detection of EAC, and 38%–56% believed that EAC screening is cost-effective (Kolb et al., 2022, Lin et al., 2002). Two surveys reported that clinicians’ belief in EAC screening benefits is low compared with colorectal cancer screening (Kolb et al., 2022, Rubenstein et al., 2008). Reported drivers for EAC screening included: ‘patient has risk factors’, ‘patient request’, ‘prior malpractice suits’, and ‘procedure reimbursement’ (Boolchand et al., 2006, Chey et al., 2005, Kolb et al., 2022, Lin et al., 2002, Rubenstein et al., 2008). Reported barriers included: ‘poor patient acceptance’, ‘insufficient evidence’, ‘risk of complications’, ‘difficulty identifying at-risk patients’, ‘lack of knowledge of guidelines’, ‘ineffective treatment’, ‘not my responsibility’, ‘competing concerns’, ‘insufficient clinic time’, ‘unsure about insurance coverage’, and ‘patients don’t understand’ (Boolchand et al., 2006, Kolb et al., 2022).

### 3.4 Qualitative analysis of expert opinion pieces

A summary of the framework analysis of perceived fulfilment of screening principles is provided below and in Table 3 (fulfillment of disease/condition and program/system principles) and Table 4 (fulfillment of test/intervention principles, stratified by screening test).

**Table 3:** Perceived fulfilment of screening principles within the disease/condition and program/system domains (Dobrow et al., 2018).

**Domain**	**Screening principle**	**2000**–**2010**	**2010**–**2022**	**What is needed**
**Disease/condition principles**	**1. Epidemiology of the disease or condition**	+/−	+/−	• Identification of a population for whom screening is relevant
	**2. Natural history of disease or condition and detectable preclinical stage**	–	+/−	• Understanding the natural history of BE and dysplasia in BE
	**3. Target population for screening**	–	+/−	• Accessible and complete documentation of personal risk information • Determining the target age range • Discriminative, validated, and accepted risk algorithms (including determination of risk-threshold value)
**Program/system principles**	7. Screening infrastructure*	–	+/−	• Technology to replace human efforts • High-volume test facilities • Strategy for handling downstream burden on endoscopic surveillance and treatment
	**8. Screening coordination and integration**	–	–	• Ownership of selecting, counselling, and testing screening participants
	**9. Screening acceptability and ethics**	**+/**−	+/−	• Information access for the public • Evaluation of stigmatizing effect of risk-based screening (sex, ethnicity, obesity, smoking and alcohol consumption are all socially sensitive) • Evaluation of the psychological impact of EAC screening
	**10. Screening benefits and harms**	**–**	–	• RCT level evidence on benefits and harms
	**11. Economic evaluation**	+/−	+	• Financial resources
	**12. Quality and performance management**	–	–	• Monitoring system (in the case of a screening program)

BE, Barrett’s esophagus; EAC, esophageal adenocarcinoma; RCT, randomized controlled trial.

* The test/intervention principles (4, 5 and 6) are shown in Table 4.

**Table 4:** Perceived fulfilment of screening principles within the test/intervention domains (Dobrow et al., 2018), stratified by screening tests.

				*Conventional upper endoscopy*		*Transnasal endoscopy*		*Non-endoscopic cell-collection devices*		*Analysis of circulating and exhaled biomarkers*
**Domain**	**Screening principle**			**Currently known limitations**		**Currently known limitations**		**Currently known limitations**		**Currently known limitations**
Test/intervention principles	4. Screening test performance characteristics		+/−	• Not affordable • Not efficient • Restricted to secondary care	+/−	• Low uptake • Requirement expensive equipment • Incongruent tolerability	+/−	• Limited uptake in trials • Some individuals’ inability to swallow the device • Test sensitivity	+	• Performance characteristics not validated among the target population • Test specificity
	5. Interpretation of screening test results		+	• Endoscopic and pathological interobserver variability	+	• Limited ability to identify dysplasia (small biopsy size) • Interobserver variability	+	• Chance of unequivocal test results	+/−	• Black box
	6. Post screening test options	*Diagnostic follow-up*	−	• Unclear if once-only or periodic screening is more effective • Effectiveness of endoscopic surveillance of BE as currently practiced is questionable	−	• Need for confirmatory endoscopy • Similar to conventional endoscopy (effectiveness surveillance/once-only or periodic)	−	• Need for confirmatory endoscopy • Similar to conventional endoscopy (effectiveness surveillance/once-only or periodic)	−	• Need for confirmatory endoscopy • Similar to conventional endoscopy (effectiveness surveillance/once-only or periodic)
		*Treatment*	++	• Endoscopic therapy is widely available and proven to be effective	++	• Endoscopic therapy is widely available and proven to be effective	++	• Endoscopic therapy is widely available and proven to be effective	++	• Endoscopic therapy is widely available and proven to be effective

#### 3.4.1 Principle one | epidemiology of the disease or condition

Most professionals view EAC as an important public health problem due to its exponentially rising incidence (range: 300%–700% increase in recent decades) (Blevins and Iyer, 2017, Crockett et al., 2010, di Pietro et al., 2015, Eisen et al., 2004, Falk, 2019, Fitzgerald, 2005, Gopal et al., 2004, Kolb and Wani, 2021, Lieberman and Sampliner, 2001, Malagelada, 2011, O’Donovan and Fitzgerald, 2018, Otaki and Iyer, 2018, Sami and Iyer, 2018, Sharma and Sidorenko, 2005, Spechler, 2004), late stage disease presentation (>50% presenting with stage II disease or higher) (Cook and Thrift, 2021, Crockett et al., 2010, Desai et al., 2021, di Pietro and Fitzgerald, 2013, Gopal et al., 2004, Katzka and Fitzgerald, 2020, Kolb and Wani, 2021, Lieberman and Sampliner, 2001, O’Donovan and Fitzgerald, 2018, Vaughan and Fitzgerald, 2015), and poor outcome (range: 5-year survival rate of 15–20%) (Blevins and Iyer, 2017, Cook and Thrift, 2021, di Pietro et al., 2015, Enslin and Kaul, 2020, Falk, 2019, Kolb and Wani, 2021, O’Donovan and Fitzgerald, 2018, Otaki and Iyer, 2018). However, EAC is also seen as a minor public health problem compared to other cancer types due to its relatively low absolute population risk (Blevins and Iyer, 2017, Bretthauer and Kalager, 2013, Dellon and Shaheen, 2005, Eisen et al., 2004, Fitzgerald, 2005, Kamboj et al., 2021, Lao-Sirieix and Fitzgerald, 2012, Malagelada, 2011, O’Donovan and Fitzgerald, 2018, Spechler, 2004, Spechler et al., 2018).

Some professionals see BE as an important health problem because of the increased relative risk of developing EAC (range: 30- to 125-fold greater lifetime risk) (Dellon and Shaheen, 2005, Amadi and Gatenby, 2017, Gopal et al., 2004). Others argue against this because the absolute risk for developing EAC in BE is small (range: annual risk of 0.12%–0.5%), and BE patients are more likely to die from other causes than EAC (Eisen et al., 2004, Esserman et al., 2014, Frei et al., 2021, Kamboj et al., 2021, Knox, 2011, Kuipers, 2011, Otaki and Iyer, 2018, Patel and Gyawali, 2019, Sami and Iyer, 2018, Shaheen and Palmer, 2009, Spechler, 2004). Thus, judgment on the importance of BE and EAC is equivocal.

#### 3.4.2 Principle two | natural history of disease or condition

Most professionals adhere to the following pathological sequence theory: intestinal metaplasia (IM) typical for BE developing under the influence of GERD, then developing into low-grade dysplasia (LGD) or high-grade dysplasia (HGD) in BE, and finally developing into early EAC or even advanced EAC (Dellon and Shaheen, 2005, di Pietro and Fitzgerald, 2013, Gopal et al., 2004, Kamboj et al., 2021, Patel and Gyawali, 2019, Wani and Sharma, 2006, Malagelada, 2011, Desai et al., 2021, Fitzgerald, 2005). Based on this, they see BE as a detectable preclinical phase of EAC (Amadi and Gatenby, 2017, Blevins and Iyer, 2017, Crockett et al., 2010, Dellon and Shaheen, 2005, Graham et al., 2016, Kuipers, 2011, Lao-Sirieix and Fitzgerald, 2012, Tan and di Pietro, 2022). However, some professionals perceive the pathological sequence theory as a reiterated paradigm unsupported by direct evidence (Gopal et al., 2004, Lieberman and Sampliner, 2001, Cook and Thrift, 2021). Contradicting this theory is the lack of concomitant detectable IM in surgical/biopsy specimens in a proportion of cases with EAC (range: 0%–77%) (Cook and Thrift, 2021, Dellon and Shaheen, 2005, di Pietro et al., 2015, Katzka and Fitzgerald, 2020, Vaughan and Fitzgerald, 2015). Some researchers suggest the possibility of two EAC phenotypes, one arising in a background of IM and another phenotype without the necessity of IM, (Kamboj et al., 2021, Katzka and Fitzgerald, 2020, Cook and Thrift, 2021) or that pre-existent IM is overgrown by EAC (di Pietro et al., 2015, Katzka and Fitzgerald, 2020, O’Donovan and Fitzgerald, 2018). The uncertainty about distinct EAC phenotypes limits screening and surveillance strategies that depend on BE detection (Katzka and Fitzgerald, 2020). Professionals further note that the natural history of LGD and indefinite dysplasia (IND) is insufficiently understood due to variation in reported rates for progression to HGD or EAC, or even regression to IM (Amadi and Gatenby, 2017, Eisen et al., 2004, Lieberman and Sampliner, 2001, Patel and Gyawali, 2019).

#### 3.4.3 Principle three | target population for screening

There is no consensus on the target population for EAC screening (Amadi and Gatenby, 2017, Frei et al., 2021, Tan et al., 2021, Crockett et al., 2010, Blevins and Iyer, 2017, Cook and Thrift, 2021). Professionals state that targeting GERD patients alone results in significant miss rates, because a large proportion of EAC patients (range: 40%–57%) have no history of GERD symptoms before diagnosis (Dellon and Shaheen, 2005, Shaheen et al., 2002, Amadi and Gatenby, 2017, di Pietro et al., 2015, Eisen et al., 2004, Lieberman and Sampliner, 2001, Patel and Gyawali, 2019, Rubenstein and Thrift, 2015, Atkinson and Chak, 2010, Crockett et al., 2010, de Jonge et al., 2014, Sharma and Sidorenko, 2005, Graham and Tan, 2020, Desai et al., 2021, Spechler, 2004, Shaheen and Palmer, 2009, Knox, 2011, Katzka and Fitzgerald, 2020, Kolb and Wani, 2021, Spechler et al., 2018, Tan et al., 2021, Tan and di Pietro, 2022). They think that failure to report GERD may result from experiencing atypical symptoms (e.g., cough) (Gopal et al., 2004, Patel and Gyawali, 2019, Sharma and Smith, 2020), the esophageal hyposensitivity associated with BE despite high reflux burden (Lao-Sirieix and Fitzgerald, 2012, O’Donovan and Fitzgerald, 2018, Patel and Gyawali, 2019, Rubenstein and Thrift, 2015), or not seeking help (and using over-the-counter medication) (di Pietro et al., 2015, Kamboj et al., 2021, di Pietro et al., 2018). Risk-assessment tools combining the presence of GERD with additional risk factors are seen as potentially valuable instruments to identify the at-risk population more precisely (Blevins and Iyer, 2017, Cook and Thrift, 2021, Falk, 2019, Fitzgerald, 2005, Frei et al., 2021, Graham and Tan, 2020, Kamboj et al., 2021, Kolb and Wani, 2021, O’Donovan and Fitzgerald, 2018, Tan et al., 2021, Vaughan and Fitzgerald, 2015, Yusuf and Fitzgerald, 2021). Professionals note the need to determine the target age range for screening (de Jonge et al., 2014), and, in the case of implementing a risk-assessment tool, to determine the risk threshold (Yusuf and Fitzgerald, 2021, Blevins and Iyer, 2017), evaluate acceptance among physicians and individuals (Tan and di Pietro, 2022), and incorporate family history, comorbidity, and life expectancy (Kamboj et al., 2021, Otaki and Iyer, 2018, Tan and di Pietro, 2022, Blevins and Iyer, 2017, Enslin and Kaul, 2020). There is no consensus on the benefits of screening females and non-Caucasian ethnicities (Otaki and Iyer, 2018, Rajendra, 2015, Tan et al., 2021, Tan and di Pietro, 2022).

Published ideas for retrieving personal risk information include: 1) targeting acid suppression therapy users through electronic patient files in primary care (O’Donovan and Fitzgerald, 2018), 2) applying a risk calculator on these files (Vaughan and Fitzgerald, 2015), or 3) applying web-based self-assessment risk calculators in the general population (Yusuf and Fitzgerald, 2021).

#### 3.4.4 Principle four | screening test performance characteristics

Conventional EGD is not considered an option for widespread screening due to its high cost, risk of adverse events, and use limited to secondary care (Atkinson and Chak, 2010, Crockett et al., 2010, Cross, 2011, Dellon and Shaheen, 2005, Kamboj et al., 2021, Kuipers, 2011, Lao-Sirieix and Fitzgerald, 2012, O’Donovan and Fitzgerald, 2018, Sami and Iyer, 2018, Spechler, 2004, Yusuf and Fitzgerald, 2021).

The fact that TNE can be performed outside the hospital, which allows higher throughput compared with sedated endoscopy, is viewed as an advantage (Craanen and Kuipers, 2001, Crockett et al., 2010, O’Donovan and Fitzgerald, 2018, Patel and Gyawali, 2019, Spechler et al., 2018, Tan and di Pietro, 2022). However, uptake by physicians and the public is low, likely due to the requirement of professional expertise and expensive equipment (less applicable in primary care), small biopsy size, and incongruent patient tolerability and preference (di Pietro et al., 2015, di Pietro et al., 2018, Frei et al., 2021, Lao-Sirieix and Fitzgerald, 2012, O’Donovan and Fitzgerald, 2018, Sami and Iyer, 2018, Tan et al., 2021, Tan and di Pietro, 2022).

Video capsule endoscopy is not viewed as a suitable technique for screening. This is due to the time-intensive need for image interpretation, reduced image quality, risk of insufficient esophageal imaging or capsule retention, and inability to take biopsies (di Pietro et al., 2015, Michalak et al., 2009, Sami and Iyer, 2018, Spechler et al., 2018, Lao-Sirieix and Fitzgerald, 2012, Yusuf and Fitzgerald, 2021).

Non-endoscopic cell-collection devices, such as the Cytosponge-TFF3 or EsoCheck, are considered as safe, simple, tolerable and affordable alternatives to endoscopy that may be suitable for primary care and automated reading (di Pietro et al., 2015, Frei et al., 2021, Lao-Sirieix and Fitzgerald, 2012, O’Donovan and Fitzgerald, 2018, Spechler et al., 2018, Tan and di Pietro, 2022, Yusuf and Fitzgerald, 2021). Some professionals see these tests as a potential solution for sampling bias in endoscopy (Amadi and Gatenby, 2017). Professionals indicated that there is room for improvement in test uptake and in the addition of risk stratification biomarkers (di Pietro et al., 2015, Frei et al., 2021, Kolb and Wani, 2021, Tan et al., 2021, Lao-Sirieix and Fitzgerald, 2012, Yusuf and Fitzgerald, 2021). Potential barriers are the need for confirmatory endoscopy, poor DNA yield in some cases, limited test sensitivity, and some individuals’ inability to swallow these devices (di Pietro et al., 2015, Lao-Sirieix and Fitzgerald, 2012, Sami and Iyer, 2018, Sharma and Smith, 2020, Yusuf and Fitzgerald, 2021).

Analysis of circulating and exhaled biomarkers is seen as ideal for screening in terms of safety, tolerability, cost, and applicability in primary care (Amadi and Gatenby, 2017, Konda and Souza, 2019, Lao-Sirieix and Fitzgerald, 2012, O’Donovan and Fitzgerald, 2018, Sami and Iyer, 2018, Tan et al., 2021, Yusuf and Fitzgerald, 2021). However, professionals note that external validation and reproducibility of conducted studies is challenging (Frei et al., 2021, Tan et al., 2021, Tan and di Pietro, 2022), and they view the need for confirmatory endoscopy as disadvantageous (O’Donovan and Fitzgerald, 2018, Spechler et al., 2018, Yusuf and Fitzgerald, 2021).

#### 3.4.5 Principle five | interpretation of screening test results

In the case of non-endoscopic cell-collection devices, it is noted that decreasing the number of equivocal test results and defining a follow-up strategy for patients with low-confidence results is needed to achieve this principle (Kolb and Wani, 2021).

Regarding conventional EGD, professionals are concerned that interobserver variability (endoscopic and pathologic) may result in widespread false-positive BE diagnosis. Perceived causes include accidental biopsy of IM at the gastro-esophageal junction (Dellon and Shaheen, 2005, Kolb and Wani, 2021, Crockett et al., 2010, Falk, 2019, Gerson, 2011, de Jonge et al., 2014, Graham et al., 2016), or reluctance to accept the 1 cm threshold for BE diagnosis (Kamboj et al., 2021, Falk, 2019). Additionally, histopathological interobserver variability may affect LGD diagnosis and the distinction between HGD and early EAC, especially when erosive esophagitis is present (Dellon and Shaheen, 2005, Patel and Gyawali, 2019, Wani and Sharma, 2006, Craanen and Kuipers, 2001, Crockett et al., 2010, Sharma and Sidorenko, 2005, Malagelada, 2011, Spechler, 2004, Blevins and Iyer, 2017, Shaheen and Palmer, 2009). Misclassification of BE and dysplasia may lead to oversurveillance and overtreatment (Crockett et al., 2010). Assessment by a specialized gastrointestinal pathologist is perceived as helpful in preventing misclassification (Patel and Gyawali, 2019, Falk, 2019).

#### 3.4.6 Principle six | post screening test options

There is no consensus on whether once-only or periodic screening has the best benefit-harm ratio. Professionals reason that BE developing after the age of 50 years is unlikely to progress to EAC within the remainder of the patient’s life (Gopal et al., 2004, Falk, 2019). They therefore suggest that, if screening aims to detect BE, once-only screening may be appropriate. However, it would be optimal if this initial test would, next to proving the presence or absence of BE, also allow for stratification in low- and high-risk for progression groups (Frei et al., 2021). Screening aimed at detecting dysplasia and early EAC may replace endoscopic surveillance and should be offered periodically (Kolb and Wani, 2021).

Regarding endoscopic follow-up, the effectiveness of current surveillance programs is considered questionable (di Pietro et al., 2015, Eisen et al., 2004, Frei et al., 2021, Malagelada, 2011, O’Donovan and Fitzgerald, 2018, Sharma and Sidorenko, 2005, Vaughan and Fitzgerald, 2015). Professionals highlight the lack of RCTs reporting EAC-specific mortality reduction following BE surveillance (di Pietro and Fitzgerald, 2013, Gopal et al., 2004, Otaki and Iyer, 2018, Atkinson and Chak, 2010, Knox, 2011, Bretthauer et al., 2016), and the many biases in observational surveillance studies (di Pietro and Fitzgerald, 2013, Shaheen et al., 2002, Spechler, 2004, Spechler et al., 2018).

Regarding treatment options, professionals agree that evidence sufficiently supports the effectiveness of endoscopic therapy for treatment of early-stage EAC (stage T1a) and prevention of progression of dysplastic BE (Blevins and Iyer, 2017, di Pietro et al., 2015, di Pietro et al., 2018, di Pietro and Fitzgerald, 2013, Kamboj et al., 2021, Kolb and Wani, 2021, Lao-Sirieix and Fitzgerald, 2012, O’Donovan and Fitzgerald, 2018, Otaki and Iyer, 2018, Patel and Gyawali, 2019, Sami and Iyer, 2018, Tan and di Pietro, 2022, Vaughan and Fitzgerald, 2015). This is in contrast to the situation before 2010, when esophagectomy was the only treatment option for both HGD and EAC (Lieberman and Sampliner, 2001, Shaheen and Palmer, 2009, Knox, 2011).

#### 3.4.7 Principle seven | infrastructure

Professionals’ main concern is that systematically inviting individuals for EAC screening would drain medical resources, i.e., trained physicians/assistants for triage and performing the screening test, equipment, and, depending on the test used, pathology services (Cook and Thrift, 2021, Craanen and Kuipers, 2001, Crockett et al., 2010, de Jonge et al., 2014, Eisen et al., 2004, Gopal et al., 2004, Katzka and Fitzgerald, 2020, Lao-Sirieix and Fitzgerald, 2012, Vaughan and Fitzgerald, 2015). Additionally, downstream confirmatory endoscopy, surveillance, and treatment services would require experienced and advanced endoscopists, expert pathologists, anesthetists, endoscopy and recovery rooms, nurses, planners, and equipment (Cook and Thrift, 2021, Craanen and Kuipers, 2001, Crockett et al., 2010, de Jonge et al., 2014, Eisen et al., 2004, Gopal et al., 2004, Katzka and Fitzgerald, 2020, Lao-Sirieix and Fitzgerald, 2012, Vaughan and Fitzgerald, 2015). Professionals suggest that technological developments might facilitate initial screening test logistics (e.g., sample-tracking), remote pathologist reporting (e.g., slide-scanner technology and machine learning assistance), and communication (e.g., electronic reporting systems that include recommendations for management) (Frei et al., 2021, O’Donovan and Fitzgerald, 2018, Tan and di Pietro, 2022). They note that these reporting systems must ensure patient confidentiality and data protection (O’Donovan and Fitzgerald, 2018). Depending on the screening test, mass transportation and storage of specimens may also be needed (O’Donovan and Fitzgerald, 2018).

#### 3.4.8 Principle eight | coordination and integration

According to some professionals, a formal system is needed to inform, invite, counsel, test, and manage the treatment of screening participants (Bretthauer et al., 2016, Ilbawi and Anderson, 2015). Although several countries already have screening organizations in place, it is unclear whether these organizations should be responsible for potential implementation and monitoring of EAC screening as this depends on the target population and country-specific legislation (Kolb and Wani, 2021). Furthermore, some authors of included articles assumed that PCPs would take ownership of selecting, counseling and, in the case of non-endoscopic cell-collection devices*,* testing participants (Cross, 2011, Kamboj et al., 2021, Smith et al., 2015). However, it is not evident that all PCPs will support EAC screening (Boolchand et al., 2006).

#### 3.4.9 Principle nine | ethics and risk communication

Professionals have identified several ethical concerns, including potential insurance discrimination following BE diagnosis (particularly in the US) (Crockett et al., 2010, de Jonge et al., 2014, Dellon and Shaheen, 2005, Falk, 2019, Gerson, 2011, Kuipers, 2011, Shaheen and Palmer, 2009, Sharma and Sidorenko, 2005), and false reassurance for individuals due to false-negative test results (Craanen and Kuipers, 2001, Lao-Sirieix and Fitzgerald, 2012). Other identified ethical implications concern the attitude of health care providers. The idea that screening can be harmful is counterintuitive, which may be dangerous if screening is causing more harm than good (Bretthauer and Kalager, 2013, Esserman et al., 2014). Providers may also be driven to perform screening for reasons other than belief in effectiveness, such as a fee-for-service model, fear of liability claims, the expectation that risk communication requires more time than esophageal examination, patient request, fear of missing cancer, or frustration about the inability to reduce EAC mortality (Adams et al., 2014, Crockett et al., 2010, Esserman et al., 2014, Shaheen, 2011, Zakko et al., 2017). Furthermore, inviting individuals based on sex may be seen as sexist (Shaheen, 2011).

Moreover, professionals report that education about EAC risk may lead to cancer worry (Eisen et al., 2004, de Jonge et al., 2014, Lao-Sirieix and Fitzgerald, 2012, Sharma and Sidorenko, 2005, Graham et al., 2016, Esserman et al., 2014). This may increase if individuals undertake an online search, which is likely to suggest an unreasonable high cancer risk (Crockett et al., 2010, de Jonge et al., 2014, Gerson, 2011, Kuipers, 2011). It is therefore recommend to train physicians in careful risk communication at all stages of the screening process (Graham et al., 2016, Cook and Thrift, 2021, Mehta and Asch, 2014). Professionals further suggest reframing non-dysplastic BE as an alteration of tissue, and framing LGD and HGD in BE as precancerous (Esserman et al., 2014, Mehta and Asch, 2014).

#### 3.4.10 Principle ten | benefits and harms

**Fig. 2:**
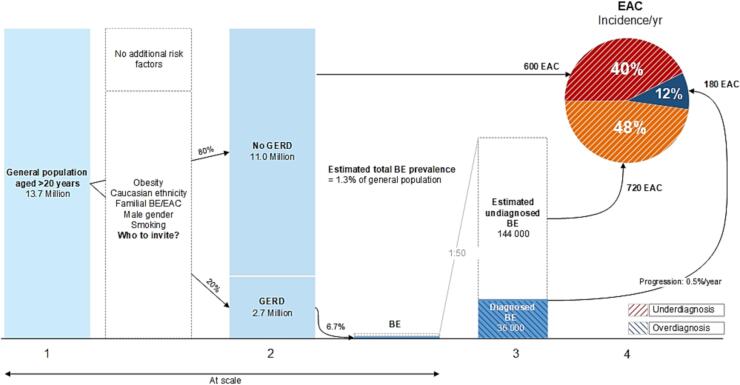
Under- and overdiagnosis in the current esophageal adenocarcinoma screening and surveillance paradigm. Legend: 1. Dutch population in 2021. 2. Based on systematic literature review, GERD symptoms are prevalent in 20% of the general population (El-Serag et al., 2014). 3. Calculated from estimated EAC cases (see number 4) and a BE to EAC progression rate of 0.5%/year (Peters et al., 2019). Prior BE diagnosis: 36 000 × 0.5%/year = 180 EAC/year. Estimated undiagnosed BE: 144 000 × 0.5%/year = 720 EAC/year. The calculated total BE prevalence of 180 000 (1.3% of the general population; 6.7% of the population with GERD) is in line with a meta-analysis reporting global BE prevalence of 0.96% and prevalence of BE in GERD of 6.7% (Marques de Sa et al., 2020). 4. There were 2500 esophageal cancer cases in 2021 in the Netherlands, of which approximately 1500 (61%) were adenocarcinomas (Vegt et al., 2022). Based on a meta-analysis, an estimated 180 cases (12%) had prior BE diagnosis (Tan et al., 2020). Approximately 600 (40%) had no prior BE diagnosis and no history of GERD symptoms (Chak et al., 2006). The remaining 720 cases (48%) had history of GERD symptoms without prior BE diagnosis.

According to professionals, this principle is not fulfilled due to the lack of RCTs on benefits and harms of EAC screening (Bretthauer and Kalager, 2013, Craanen and Kuipers, 2001, Crockett et al., 2010, Eisen et al., 2004, Gerson, 2011, Kolb and Wani, 2021, Kuipers, 2011, Malagelada, 2011, Michalak et al., 2009, Otaki and Iyer, 2018, Rajendra, 2015, Reid, 2017, Sami and Iyer, 2018, Shaheen et al., 2009, Sharma and Sidorenko, 2005, Wani and Sharma, 2006, Zakko et al., 2017). Perceived potential benefits of screening include the increased opportunity for early diagnosis and curative treatment (di Pietro et al., 2015, Sami and Iyer, 2018, Spechler, 2004, Shaheen and Palmer, 2009), the theoretical ability to decrease EAC-related mortality (di Pietro et al., 2015, Falk, 2002, Gopal et al., 2004, Lao-Sirieix and Fitzgerald, 2012, Michalak et al., 2009, O’Donovan and Fitzgerald, 2018, Patel and Gyawali, 2019, Sami and Iyer, 2018, Spechler, 2004), reassurance for participants following a negative test result (Spechler, 2004), and the potential quality-of-life (QoL) benefit following early treatment compared with esophagectomy for advanced EAC (Otaki and Iyer, 2018). Reported potential harms of EAC screening include iatrogenic injury (Crockett et al., 2010, Cross, 2011, de Jonge et al., 2014, Dellon and Shaheen, 2005, Eisen et al., 2004, Enslin and Kaul, 2020, Kamboj et al., 2021, Knox, 2011, Kuipers, 2011, Malagelada, 2011, O’Donovan and Fitzgerald, 2018, Sami and Iyer, 2018, Spechler, 2004, Spechler et al., 2018), psychological distress (Kolb and Wani, 2021, Craanen and Kuipers, 2001, Lao-Sirieix and Fitzgerald, 2012, Sharma and Sidorenko, 2005, Bretthauer and Kalager, 2013, Gerson and Triadafilopoulos, 2002), false-positive or -negative test results (Kolb and Wani, 2021, Lieberman and Sampliner, 2001, Craanen and Kuipers, 2001, Lao-Sirieix and Fitzgerald, 2012, Sharma and Sidorenko, 2005, Bretthauer and Kalager, 2013), and decreased QoL due to a BE diagnosis (Bretthauer and Kalager, 2013, Crockett et al., 2010, de Jonge et al., 2014, Eisen et al., 2004, Kuipers, 2011). Professionals are concerned that overdiagnosis, detecting conditions that may never cause symptoms during a lifetime, will expose the target population to additional harm (Bretthauer et al., 2016, Bretthauer and Kalager, 2013, Craanen and Kuipers, 2001, Ilbawi and Anderson, 2015, Lambert, 2012, Reid, 2017, Sharma and Sidorenko, 2005, Vaughan and Fitzgerald, 2015). There is also concern that underdiagnosis, missing cancers that then present at advanced stages in the clinic, may diminish screening benefit (Fig. 2) (Reid, 2017, Sami and Iyer, 2018).

#### 3.4.11 Principle eleven | economic evaluation

EAC screening is expected to require significant financial resources (Bretthauer et al., 2016, Cook, 2013, Craanen and Kuipers, 2001, di Pietro and Fitzgerald, 2013, Falk, 2019, Frei et al., 2021, Gopal et al., 2004, Kamboj et al., 2021, Katzka and Fitzgerald, 2020, Knox, 2011, Kuipers, 2011, Lao-Sirieix and Fitzgerald, 2012, Otaki and Iyer, 2018, Patel and Gyawali, 2019, Sami and Iyer, 2018, Sharma and Sidorenko, 2005, Sharma and Smith, 2020, Spechler, 2004). On the other hand, costs associated with the care of a patient with advanced esophageal cancer, such as surgery, chemotherapy, radiotherapy, hospitalizations, and cancer nursing, may be avoided (Gopal et al., 2004, Sharma and Sidorenko, 2005, Sharma and Smith, 2020). Professionals further warn for unrealistic assumptions in cost-effectiveness studies that address endoscopic screening (which show incremental cost-effectiveness ratios ranging from $10,000 to $24,000 per quality-adjusted life year), such as overestimated cancer progression rates, optimistic assumptions of participation rates and performance of screening tests, and neglecting downstream surveillance and treatment costs (Atkinson and Chak, 2010, Dellon and Shaheen, 2005, di Pietro et al., 2015, Falk, 2002, Otaki and Iyer, 2018, Spechler, 2004).

#### 3.4.12 Principle twelve | quality and performance management

The expected increase in the quality of BE management following the introduction of a potential screening program is viewed as a beneficial side-effect (Bretthauer and Kalager, 2013, Ilbawi and Anderson, 2015). Professionals further highlight the need for a system that monitors program quality and mortality if screening is implemented nationally (Bretthauer et al., 2016, Bretthauer and Kalager, 2013, Ilbawi and Anderson, 2015, Lao-Sirieix and Fitzgerald, 2012, O’Donovan and Fitzgerald, 2018).

### 3.5 Screening recommendations over time

**Fig. 3:**
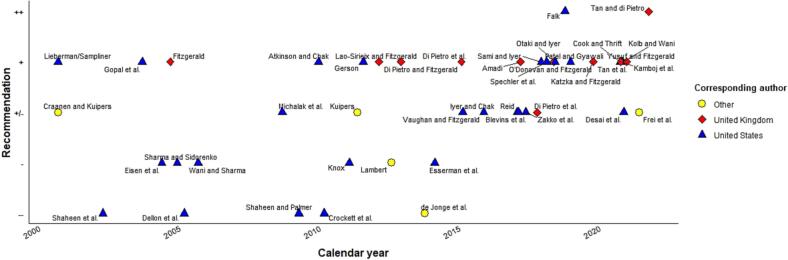
Professionals’ recommendations regarding targeted screening for esophageal adenocarcinoma. Legend*:* ++, recommending; +, motivation; +/−, neutral position; −, serious doubt; −/−, recommending against.

**Fig. 4:**
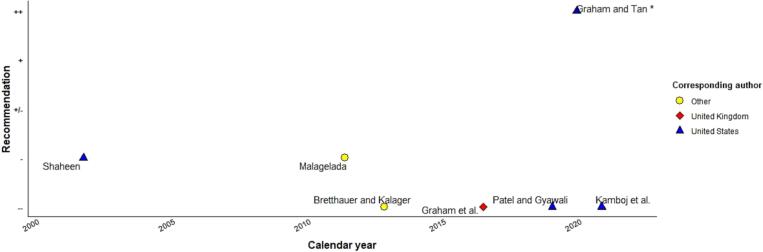
Professionals’ recommendations regarding population screening for esophageal adenocarcinoma. Legend*:* ++, recommending; +, motivation; +/−, neutral position; −, serious doubt; −/−, recommending against.

Screening recommendations were extracted from 49 expert opinion articles (text fragments and blinded categorization results are available in Supplementary Table 3). In Fig. 3, professionals’ recommendations regarding targeted EAC screening are mapped by calendar year (substantial inter-rater agreement κ_w_ = 0.71; SE = 0.062), showing that their attitudes towards targeted screening appear increasingly supportive. In contrast, Fig. 4 depicting professionals’ attitude toward population screening for EAC is mostly negative (almost perfect inter-rater agreement κ_w_ = 0.87; SE = 0.144).

## 4 Discussion

Our findings imply a discrepancy between professionals’ increased support for targeted EAC screening and the continued perceived unfulfillment of most screening principles. It appears that professionals’ motivation is mainly driven by the recent introduction of novel less-invasive screening modalities and the possibility to safely and effectively treat screen-detected neoplasia. However, examination of professionals’ perceptions reveals two major bottlenecks preventing full acceptance of EAC screening: the unmet need to precisely identify a target population with a high burden of EAC and the lack of favorable data on screening benefits and harms. We further identified several surmountable implications, such as the need to determine how often to screen, which screening test to use, how to manage non-dysplastic BE, how to inform the public, and how to adequately invest human and financial resources (see Table 3 for future research directions).

Although most professionals agree that the burden of EAC does not merit population screening, their belief in the possibility of identifying subpopulations at risk of developing EAC appears to have grown over the last decade. The increased interest in screening for relatively low-incident cancers aligns with the ‘new EU approach on cancer screening’ released by the European Health Union, September 2022 (European Commision, 2022). This new approach aims to increase the number of screenings, covering more target groups and more cancers. Similar to the findings presented in this review, the EU proposal refers to the promise of risk-assessment tools to optimize risk-based cancer screening. Implementation of these tools will rely on discriminatory accuracy, transparency and validation of the model, provider and public acceptance, and definition of criteria for sufficient evidence to declare a model as ‘valid’ for the selection of individuals at high risk for EAC (Shaheen et al., 2016, Eddy et al., 2012).

Our analysis further revealed that randomized studies on screening benefits and harms are crucial to establish professionals’ full support for EAC screening. Despite numerous studies on the feasibility, safety, acceptability, and efficacy of several BE screening devices, only two studies have, to the best of our knowledge, addressed the effectiveness of BE screening. One multicenter, pragmatic, randomized, controlled trial showed that the Cytosponge-TFF3 technology detected ten times more cases of BE compared with usual care (Fitzgerald et al., 2020). Another targeted screening trial, which is expected to finish in 2035, aims to determine the extent to which the Cytosponge-TFF3 can reduce mortality from EAC (BEST4).

Evaluation of the long-term effect of screening requires sufficient follow-up time because the impact of screening on mortality reduction takes at least ten years to become evident. This is challenging because rapidly developing technologies (risk-assessment tools, screening modalities, artificial intelligence models to assess collected samples, and biomarker panels to risk stratify them) may diminish the relevance of screening trials that are started now. In addition, variation in these elements and screen intervals, start- and stop ages, and public participation can change the benefit-harm trade-offs (Albhert et al., 2017). For similar reasons, breast and bowel cancer experts recommend hybrid effectiveness-implementation research combined with modeling studies to evaluate the long-term population outcomes of risk-stratified cancer screening (Pashayan et al., 2020, Green et al., 2019). It is advisable that researchers intending to set up an EAC screening trial collaborate with policy makers to determine if such outcome measures would deliver sufficient proof of effectiveness.

The strengths of this literature review include the rigorous qualitative analysis of expert opinion articles and the application of a theoretical framework. Our method is also highly transparent since the included articles, i.e., the data supporting the findings, are publicly available and free from privacy restrictions. However, the following limitations should be taken into consideration. First, original peer-reviewed studies reporting professionals’ views on EAC screening were limited to the US, mainly published before 2010, and only addressed the use of endoscopy for this purpose. Surveys conducted in other contexts may yield different estimates of clinicians’ belief in (cost-) effectiveness and drivers/barriers to screening. Second, the opinions of professionals summarized in this review are not static and will likely shift when results of new studies become available. Similarly, few included articles contain reflections on the most recent innovations and epidemiologic data. Third, this review does not provide a complete overview of cost-effectiveness studies and screening or risk-stratification technologies. We refer to articles containing the authors’ reflections on novel technologies rather than the original studies reporting their performance. Fourth, the distinction between recommendation categories in Fig. 3, Fig. 4 was a matter of interpretation. Categorization was done independently by two researchers that were blinded for author and publication year of the extracted recommendations to minimize subjectivity.

### 4.1 Conclusion

Although professionals’ motivation to conduct targeted EAC screening appears to have increased over the past decade, relevant screening principles remain insufficiently fulfilled until now. In particular, the identification of an appropriate screening policy and evidence that screening reduces EAC-related mortality are still considered inadequate.

## 5 Grant support

This study was funded by the Netherlands Organization for Health Research and Development (ZonMw) under grant 555004206.

## 6 Role of the funding source

The funder of the study had no role in study design, data collection, data analysis, data interpretation, or writing of the report.

## 7 Data transparency statement

Study data are available on https://doi.org/10.17026/dans-x67-9bzx.

## Author contributions

YP, LR, MB and PS were involved in conception of the work and acquisition of funding. JS, YP, LR, MB and PS designed the study and wrote the protocol. JS and MG did the study search, study selection, data extraction, and data analysis. YP, LR, MB and PS supervised all the steps in the review process. All authors contributed to interpretation of the findings and revision of the manuscript.

## Declaration of Competing Interest

The authors declare the following financial interests/personal relationships which may be considered as potential competing interests: JS, YP, LR, MG, and MB have no conflicts of interest or financial ties related to this work to disclose. PS is receiving unrestricted research grants from Pentax (Japan), Norgine (UK), Motus GI (USA), MicroTech (China) and The eNose Company (Netherlands) and is in the advisory board of Motus GI (US) and Boston Scientific (US).

## Data Availability

We have shared the URL to the data.
