# Supplementary material for: Professionals’ views on the justification for esophageal adenocarcinoma screening: A systematic literature search and qualitative analysis
Source: Prev Med Rep. 2023 May 26;34:102264. doi: 10.1016/j.pmedr.2023.102264 (PMC10236474; doi:10.1016/j.pmedr.2023.102264)
Supplement: Supplementary data 1 [file mmc1.docx]

**Supplemental methods**

*Calculating interrater-agreement*

For assessing inter-rater agreement beyond chance on the selection of articles and the categorization of professionals’ general opinions, we used SPSS (version 25; IBM Corporation, Armonk, NY) to calculate Cohen’s kappa (κ) values. We calculated weighted kappa coefficients (κw) in the linear model for ordinal data. A κ-value of more than 0.8, more than 0.6, and more than 0.4 is considered to have “almost perfect,” “substantial,” and “moderate” agreement, respectively.

**Supplementary table 1.** Search strategies

| **Ovid MEDLINE(R) ALL**1946 to April 01, 2021  1. Esophageal Neoplasms/  2. ((esophag$ or oesophag$) adj3 (neoplas$ or cancer$ or tumo?r or carcinoma$ or malignan$ or oncolog$ or adenocarcinoma$ or adeno-carcinoma$)).tw,kw.  3. Barrett Esophagus/  4. (Barret$ adj1 (esophag$ or oesophag$ or epitheli$ or metaplasi$ or syndrome?)).tw,kw.  5. ((esophag$ or oesophag$) adj3 (dysplasia or dysplastic$ or precancer$ or pre-cancer$ or premalignant$ or pre-malignan$)).tw,kw.  6. 1 or 2 or 3 or 4 or 5  7. Mass screening/  8. Early detection of cancer/  9. exp Preventive Medicine/  10. exp Preventive Health Services/  11. "referral and consultation"/ or gatekeeping/ or physician self-referral/  12. (screening$ or screened or screen or prevent$).tw,kw.  13. (early adj3 detect$).tw,kw.  14. 7 or 8 or 9 or 10 or 11 or 12 or 13  15. attitude to health/ or health knowledge, attitudes, practice/  16. "delivery of health care"/ or health services accessibility/ or healthcare disparities/ or practice patterns, physicians'/  17. Decision Making, Shared/  18. (acceptab$ or acceptance or adhere$ or approach$ or attitude$ or barrier$ or balancing or belief$ or challenge$ or controversie$ or compliance or comply or choice or decide$ or decision$ or facilit$ or framework or incentive$ or intention$ interest$ or opinion$ or paradigm or professional perspective$ or perception$ or (perceive$ adj2 (threat$ or risk$)) or practice or prefer$ or rationale or (risk adj 1 assess$) or scope or select or willing$ or worrie$ or worry$).tw,kw.  19. (against or time or ready or far or future or motion or pro or con or reality).ti.  20. Ethnic Groups/  21. exp ethics/  22. ethics.fs.  23. exp Jurisprudence/  24. Legislation & Jurisprudence.fs.  25. Public Opinion/  26. psychology.fs.  27. "Cost Savings"/  28. exp "Costs and Cost Analysis"/  29. (access$ or acculturation or administrative or autonomy or community health aid$ or communicat$ or cost$ or culture$ or ethic$ or financ$ or implementation or implication$ or infrastructure or insurance or legal$ or logistical or motivat$ or organizat$ or psychol$ or public health or reimbursement or religion or resource$ or start-up or stigma or unavailab$).tw,kw.  30. ((cost adj1 effect$) or cost-effect$ or cost-utility or economi$).tw,kw.  31. 15 or 16 or 17 or 18 or 19 or 20 or 21 or 22 or 23 or 24 or 25 or 26 or 27 or 28 or 29 or 30  32. Qualitative research/  33. Interview, Psychological/ or Interview/  34. Focus groups/  35. Health Care Surveys/  36. Questionnaires/  37. ((semi-structured or semistructured or unstructured or informal or in-depth or indepth or structure$ or guide$) and (interview$ or discussion$ or question?aire$)).tw,kw.  38. 32 or 33 or 34 or 35 or 36 or 37  39. 31 or 38  40. 6 and 14 and 39  41. limit 40 to yr="2000 -Current"  **Hits: 2351**  **Hits search update 22 September 2022: 480** |
| --- |
| **Embase**1974 to 2021 April 01  1. esophagus tumor/ or esophagus cancer/  2. ((esophag$ or oesophag$) adj3 (neoplas$ or cancer$ or tumo?r or carcinoma$ or malignan$ or oncolog$ or adenocarcinoma$ or adeno-carcinoma$)).tw,kw.  3. Barrett Esophagus/  4. (Barret$ adj1 (esophag$ or oesophag$ or epitheli$ or metaplasi$ or syndrome?)).tw,kw.  5. ((esophag$ or oesophag$) adj3 (dysplasia or dysplastic$ or precancer$ or pre-cancer$ or premalignant$ or pre-malignan$)).tw,kw.  6. 1 or 2 or 3 or 4 or 5  7. Mass screening/  8. early cancer diagnosis/  9. exp preventive medicine/  10. exp patient referral/  11. preventive health service/  12. (screening$ or screened or screen or prevent$).tw,kw.  13. (early adj3 detect$).tw,kw.  14. 7 or 8 or 9 or 10 or 11 or 12 or 13  15. exp attitude to health/  16. Physician's Practice Patterns/  17. Guideline Adherence/  18. (acceptab$ or acceptance or adhere$ or against or approach$ or attitude$ or barrier$ or balancing or belief$ or challenge$ or controversie$ or compliance or comply or choice or decide$ or decision$ or facilit$ or framework or incentive$ or insight or intention$ interest$ or management or opinion$ or option$ or paradigm or professional perspective$ or perception$ or (perceive$ adj2 (threat$ or risk$)) or practice or prefer$ or rationale or risk assess$ or scope or select or willing$ or worrie$ or worry$).tw,kw.  19. (time or ready or far or future or motion or pro or con or reality).ti.  20. exp cultural factor/  21. exp ethics/  22. legal aspect/  23. cost savings/  24. "cost-benefit analysis"/  25. (access$ or administrative or autonomy or availab$ or contract$ or community health aid$ or communicat$ or cost$ or culture$ or ethic$ or financ$ or implementation or implication$ or infrastructure or legal$ or logistic$ or motivat$ or organizat$ or psychol$ or public health or reimbursement or resource$ or start-up or unavailab$).tw,kw.  26. ((cost adj1 effect$) or cost-effect$ or cost-utility or economi$).tw,kw.  27. 15 or 16 or 17 or 18 or 19 or 20 or 21 or 22 or 23 or 24 or 25 or 26  28. Qualitative research/  29. Interview, Psychological/ or Interview/  30. Focus groups/  31. Health Care Surveys/  32. Questionnaires/  33. ((semi-structured or semistructured or unstructured or informal or in-depth or indepth or structure$ or guide$) and (interview$ or discussion$ or question?aire$)).tw,kw.  34. 28 or 29 or 30 or 31 or 32 or 33  35. 27 or 34  36. 6 and 14 and 35  37. limit 36 to conference abstract  38. 36 not 37  39. limit 38 to yr="2000 -Current"  **Hits: 4423**  **Hits search update 22 September 2022: 821** |
| **APA PsycInfo**1806 to March Week 5 2021  1. exp Esophagus/ and exp Neoplasms/  2. ((esophag$ or oesophag$) adj3 (neoplas$ or cancer$ or tumo?r or carcinoma$ or malignan$ or oncolog$ or adenocarcinoma$ or adeno-carcinoma$)).mp.  3. (Barret$ adj1 (esophag$ or oesophag$ or epitheli$ or metaplasi$ or syndrome?)).mp.  4. ((esophag$ or oesophag$) adj3 (dysplasia or dysplastic$ or precancer$ or pre-cancer$ or premalignant$ or pre-malignan$)).mp.  5. 1 or 2 or 3 or 4  6. screening/  7. cancer screening/  8. preventive health services/  9. (screening$ or screened or screen or prevent$).mp.  10. (early adj3 detect$).mp.  11. (breath analy$ or (capsule adj1 endoscopy) or (conscious adj1 sedation) or cytosponge$ or cytodiagnos$ or endoscop$ or esophagoscop$ or gastroscop$).mp.  12. 6 or 7 or 8 or 9 or 10 or 11  13. exp health attitudes/  14. exp Social Acceptance/  15. health care delivery/  16. decision making/  17. (acceptab$ or acceptance or adhere$ or approach$ or attitude$ or barrier$ or balancing or belief$ or challenge$ or controversie$ or compliance or comply or choice or decide$ or decision$ or facilit$ or framework or incentive$ or intention$ interest$ or opinion$ or paradigm or professional perspective$ or perception$ or (perceive$ adj2 (threat$ or risk$)) or practice or prefer$ or rationale or (risk adj 1 assess$) or scope or select or willing$ or worrie$ or worry$).mp.  18. "racial and ethnic groups"/  19. Ethics/  20. exp "law (government)"/  21. Public Opinion/  22. exp Health Care Psychology/  23. health care costs/  24. mental health stigma/  25. health disparities/  26. (access$ or acculturation or administrative or autonomy or community health aid$ or communicat$ or cost$ or culture$ or ethic$ or financ$ or implementation or implication$ or infrastructure or insurance or legal$ or logistical or motivat$ or organizat$ or psychol$ or public health or reimbursement or religion or resource$ or start-up or stigma or unavailab$).mp.  27. 13 or 14 or 15 or 16 or 17 or 18 or 19 or 20 or 21 or 22 or 23 or 24 or 25 or 26  28. Qualitative research/  29. interviews/ or focus group interview/ or psychodiagnostic interview/ or semi-structured interview/  30. focus group/  31. surveys/  32. Questionnaires/  33. ((semi-structured or semistructured or unstructured or informal or in-depth or indepth or structure$ or guide$) and (interview$ or discussion$ or question?aire$)).mp.  34. 28 or 29 or 30 or 31 or 32 or 33  35. 27 or 34  36. 5 and 12 and 35  **Hits: 25**  **Hits search update 22 September 2022: 0** |

**Supplementary table 2.** Main characteristics of the included articles.

| **Original (n=5)** | | | | | |
| --- | --- | --- | --- | --- | --- |
| **Reference** | **Country** | **Study design** | **Sample size** | **Profession** | **Construct** |
| Boolchand et al., 2006^1^ | US | Cross-sectional survey | 544 | Primary care physician (n = 271) Internist (n = 215) Other (n = 56) | Barriers to BE screening and the interest in performing unsedated endoscopy |
| Chey et al., 2005^2^ | US | Cross-sectional survey | 1046 | Primary care physician (n = 1046) | Acceptability of screening GERD patients for BE |
| Kolb et al., 2022^3^ | US | Cross-sectional survey | 315 | Gastroenterologist (n = 120) Primary care physician (n = 195) | Compliance, practice patterns, and barriers to BE screening |
| Lin et al., 2002^4^ | US | Cross-sectional survey | 162 | Gastroenterologist (n = 162) | Practices and beliefs in managing BE |
| Rubenstein et al., 2008^5^ | US | Cross-sectional survey | 224 | Gastroenterologist (n = 224) | Incentives for BE screening |

| **Overview articles (n=63)** |  | | | |
| --- | --- | --- | --- | --- |
| **Reference** | **Country*** | **Journal** | **Article type** | **Focus** |
| Adams et al., 2014^6^ | US | JAMA | Research letter | Liability claims for omission or commission of EAC screening |
| Amadi, 2017^7^ | UK | World journal of gastroenterology | Review | Achievement of Wilson and Junger criteria for BE screening |
| Atkinson and Chak, 2010^8^ | US | Technical gastrointestinal endoscopy | Review | Implications of techniques for BE/EAC screening |
| Blevins et al., 2017^9^ | US | Gastrointestinal Endoscopy Clinics of North America | Review | Evaluation of target population for BE screening |
| Bretthauer and Kalager, 2013^10^ | Norway | British Journal of Surgery | Review | Implications of screening |
| Bretthauer et al., 2016^11^ | Norway | Endoscopy | Review | Evaluation of screening for GI cancers |
| Cook, 2013^12^ | US | The American Journal of Gastroenterology | Editorial | Predictive models for BE and EAC |
| Cook and Thrift, 2021^13^ | US | Gastrointestinal Endoscopy Clinics of North America | Review | Epidemiological implications of BE/EAC screening |
| Craanen and Kuipers, 2001^14^ | Netherlands | Best Practice & Research Clinical Gastroenterology | Review | Implications of population screening for GI cancer |
| Crockett et al., 2010^15^ | US | Clinical gastroenterology and hepatology | Clinical review | Implications of BE/EAC screening |
| Cross, 2011^16^ | US | The Nurse Practicioner | Short communication | Challenges for primary care |
| Dellon et al., 2005^17^ | US | Journal of clinical oncology | Review | Achievement of Sackett criteria for EAC screening |
| Desai et al., 2021^18^ | US | Gastrointestinal Endoscopy | Editorial | Implications of identifying target population |
| Dulai, 2002^19^ | US | Evidence-Based Gastroenterology | Selected summary and comment | Evaluation of evidence for BE/EAC screening |
| Di Pietro and Fitzgerald, 2013^20^ | UK | Gastrointestinal Endoscopy Clinics of North America | Review | Implications of BE/EAC screening |
| Di Pietro et al., 2015^21^ | UK | Gastroenterology | Review | Implications of techniques for BE/EAC screening |
| Di Pietro et al., 2018^22^ | UK | Gastroenterology | Review | Implications of BE/EAC screening |
| Eisen et al., 2004^23^ | US | Clinical Gastroenterology and Hepatology | Editorial | Problems with BE screening |
| Enslin and Kaul, 2020^24^ | US | Current gastroenterology reports | Review | Caveats in the management of the geriatric BE patient |
| Esserman et al., 2014^25^ | US | Lancet Oncology | Review | Benefits and harms of screening |
| Falk, 2002^26^ | US | Gastroenterology | Review | Rationale and implications of BE/EAC screening |
| Falk, 2019^27^ | US | The American Journal of Gastroenterology | Review | Potential improvements for BE/EAC screening |
| Fitzgerald, 2005^28^ | UK | Recent Results in Cancer Research | Review | Implications of BE/EAC screening |
| Frei et al., 2022^29^ | Netherlands | Gastrointestinal endoscopy | Editorial | Implications of BE screening |
| Gerson, 2011^30^ | US | Gastroenterology | Selected summary | Implications of BE/EAC screening |
| Gopal et al., 2004^31^ | US | Current opinion | Review | Arguments for and against BE/EAC screening |
| Graham et al., 2016^32^ | UK | Frontline Gastroenterology | Review | Implications of BE/EAC screening |
| Graham and Tan, 2020^33^ | US | Journal of Clinical Gastroenterology | Clinical review | Proposed strategies for EAC prevention |
| Ilbawi and Anderson, 2015^34^ | US | Science Translational Medicine | Commentary | Implications of prevention and early detection strategies |
| Iyer and Chak, 2016^35^ | US | Endoscopy | Editorial | Implications of transnasal endoscopy |
| de Jonge et al., 2014^36^ | Netherlands | Gut | Review | Implications of BE/EAC screening |
| Kamboj et al., 2021^37^ | US | Gastrointestinal Endoscopy Clinics of North America | Review | Rationale and challenges to BE/EAC screening |
| Katzka and Fitzgerald, 2020^38^ | US | Digestive Diseases and Sciences | Review | Controversies in BE/EAC screening |
| Knox, 2011^39^ | US | American Family Physician | Editorial | Arguments against BE screening |
| Kolb and Wani, 2021^40^ | US | Gastroenterology | Comment | Requirements for BE screening |
| Konda and Souza, 2019^41^ | US | Current Gastroenterology Reports | Review | Implications of using biomarkers for detection of BE/EAC |
| Kuipers, 2011^42^ | Netherlands | Gastroenterology & Hepatology | Q&A | Implications for BE screening |
| Lambert, 2012^43^ | France | World Journal of Gastrointestinal Endoscopy | Review | Implications of population screening for digestive cancer |
| Lao-Sirieix and Fitzgerald, 2012^44^ | UK | Nature Reviews Clinical Oncology | Review | Psychological and economic implications of esophageal cancer screening |
| Lieberman/Sampliner 2001^45^ | US | The American Journal of Managed Care | Debate and Q&A | Arguments for and against BE/EAC screening |
| Malagelada, 2011^46^ | Spain | Digestive Diseases | Review | Feasibility prevention of EAC |
| Mehta and Asch, 2014^47^ | US | Clinical gastroenterology and hepatology | Review | Behavioral implications for BE/EAC screening |
| Michalak et al., 2009^48^ | US | Current Gastroenterology Reports | Review | Implications of BE/EAC screening |
| O’Donovan and Fitzgerald, 2018^49^ | UK | Digestive Diseases and Sciences | Review | Feasibility high-volume BE screening |
| Otaki and Iyer, 2018^50^ | US | Digestive Diseases and Sciences | Review | Challenges and potential solutions EAC screening |
| Patel and Gyawali, 2019^51^ | US | Journal of Neurogastroenterology and Motility | Review | Benefit and cost of BE screening |
| Rajendra, 2015^52^ | Australia | Best Practice & Research Clinical Gastroenterology | Review | Implications of BE/EAC screening |
| Reid, 2017^53^ | US | Cellular and Molecular Gastroenterology and Hepatology | Review | Implications of BE/EAC screening |
| Rubenstein and Thrift, 2015^54^ | US | Best Practice & Research Clinical Gastroenterology | Review | Implications of selecting subjects for BE screening |
| Sami and Iyer, 2018^55^ | US | Current Treatment Options Gastroenterology | Review | Rationale and challenges of BE/EAC screening |
| Shaheen et al., 2002^56^ | US | The American Journal of Gastroenterology | Review | Achievement of Sackett criteria for EAC screening |
| Shaheen and Palmer, 2009^57^ | US | Surgical Oncology Clinics of North America | Review | Challenges to BE/EAC screening |
| Shaheen, 2011^58^ | US | The American Journal of Gastroenterology | Editorial | Epidemiological implications of BE/EAC screening |
| Sharma and Sidorenko, 2005^59^ | US | Gut | Review | Issues to BE screening |
| Smith, 2020^60^ | US | Gastroenterology & Hepatology | Q&A | Screening for BE |
| Spechler et al., 2004, 3 parts by R. Sampliner, P. Moayyedi and J. Spechler^61^ | US | The American Journal of Gastroenterology | Debate | Pro’s and con’s to screening for BE |
| Spechler et al., 2018^62^ | US | Gastroenterology | Commentary | Implications of techniques for BE/EAC screening |
| Tan et al., 2021^63^ | UK | Gastrointestinal Endoscopy Clinics of North America | Review | Progress in screening for BE |
| Tan and di Pietro, 2022^64^ | UK | Visceral Medicine | Review | Mistakes and wisdom in BE screening |
| Wani and Sharma, 2006^65^ | US | Best Practice & Research Clinical Gastroenterology | Review | Rationale and implications of BE/EAC screening |
| Vaughan and Fitzgerald, 2015^66^ | US | Nature Reviews Gastroenterology & Hepatology | Review | Challenges and strategies for EAC prevention |
| Yusuf and Fitzgerald, 2021^67^ | UK | Current Treatment Options Gastroenterology | Review | Challenges and strategies for EAC prevention |
| Zakko et al., 2017^68^ | US | The American Journal of Gastroenterology | Editorial | Bias in screening/surveillance studies |

* of the corresponding author.

**Supplementary table 3.** Extracted text units containing the authors’ overall conclusion about EAC screening and opinion categorization.

| **Artikel** | **Author(s)** | **Year** | **Country** | **Text unit** | **Targeted screening  Rater 1 (PS)** | **Targeted screening  Rater 2 (YP)** | **Targeted screening after discussion** | **Population screening rater 1 (PS)** | **Population screening rater 2 (YP)** | **Population screening after discussion** |
| --- | --- | --- | --- | --- | --- | --- | --- | --- | --- | --- |
| 1 | Adams et al. | 2014 | US | NA | NA | NA | NA | NA | NA | NA |
| 2 | Amadi | 2017 | UK | "Although endoscopy with systematic biopsy and standard pathological examination is currently the mainstay of screening and surveillance for Barrett’s oesophagus, there is still the need for a more cost-effective, less invasive, less cumbersome and more reliable way to conduct diagnosis, screening and surveillance." | +/- | + | + | NA | NA | NA |
| 3 | Atkinson and Chak | 2010 | US | "At present, we agree that screening should be considered on a case-by-case basis in older patients with chronic GERD. Patients at risk should be offered less expensive alternatives to standard sedated endoscopy where this is available. Patients with severe comorbidities who are poor surgical candidates should be excluded from screening programs. Research efforts should continue to identify those patients who would benefit most from screening and to improve technology allowing for cheaper yet effective screening techniques." | + | + | + | NA | NA | NA |
| 4 | Blevins et al. | 2017 | US | "Despite the exponential increase in the incidence of EAC and tremendous advances in the endoscopic treatment of BE-related dysplasia and early stage EAC, screening for the precursor lesion of EAC (BE) remains in evolution." | +/- | + | +/- | NA | NA | NA |
| 5 | Bretthauer and Kalager | 2012 | Other | "However, due to lack of one or several of Wilson and Junger’s basic requirements [5] (Table 1), none of these cancers [oesophageal and gastric carcinoma, and hepatocellular carcinoma] is considered a valid target for general population screening in most countries." | NA | NA | NA | -/- | - | -/- |
| 6 | Bretthauer et al. | 2016 | Other | NA | NA | NA | NA | NA | NA | NA |
| 7 | Cook | 2013 | US | NA | NA | NA | NA | NA | NA | NA |
| 8 | Cook and Thrift | 2021 | US | “Cost-effective approaches for primary and secondary prevention of EA are within our grasp, but it is imperative that we conduct larger studies with a stronger and more clinically focused statistical framework.” | + | + | + | NA | NA | NA |
| 9 | Craanen and Kuipers | 2001 | Other | "Since it is foreseen that circumstances will not change in the (near) future because the definitive study will in all probability not be carried out, because of the large number of participants needed for statistical power, the clinician has no other choice but to rely on individually tailored arguments for whether to screen/survey or not to screen/survey." | +/- | +/- | +/- | NA | NA | NA |
| 10 | Crockett et al. | 2010 | US | "At this time, there is insufficient evidence to recommend routine screening for BE or esophageal cancer in persons with GERD, even those with risk factors for BE, and, based on the evidence and guidelines discussed above, this patient would not require endoscopic screening for BE." | -/- | - | -/- | NA | NA | NA |
| 11 | Cross | 2011 | US | NA | NA | NA | NA | NA | NA | NA |
| 12 | Dellon et al. | 2005 | US | "When applying these principles of screening to Barrett’s esophagus and esophageal adenocarcinoma, a widespread screening program currently cannot be endorsed." | -/- | -/- | -/- | NA | NA | NA |
| 13 | Desai et al. | 2021 | US | "The future should help us understand the incorporation of such large-scale screening with a one-time upper endoscopy or what risk factor or factors in combination have the highest pre-test probability of having a diagnosis of BE." | +/- | +/- | +/- | NA | NA | NA |
| 14 | Di Pietro and Fitzgerald | 2013 | UK | "Endoscopic screening for BE has the potential to reduce the clinical impact of the changing epidemiology of EAC, but it is not cost-effective." | + | + | + | NA | NA | NA |
| 15 | Di Pietro et al. | 2015 | UK | "When the best screening approaches are identified, screening campaigns can be launched in countries with a high incidence of EAC." | + | + | + | NA | NA | NA |
| 16 | Di Pietro et al. | 2018 | UK | "Esophageal cancer fulfills many of the Junger criteria for screening now that endoscopic therapy is widely available and proven to be effective in curing early cancer and preventing progression of precursor lesions. However, esophageal cancer remains relatively uncommon, thus patient selection based on symptoms, family history and other predisposing risk factors is critical to improve cost-effectiveness. It is therefore imperative that the studies are performed in the relevant populations in order to avoid misleading estimates in sensitivity and specificity [148, 149]." | +/- | +/- | +/- | NA | NA | NA |
| 17 | Eisen et al. | 2004 | US | “The current recommendations for screening and surveillance to prevent death from adenocarcinoma of the esophagus are likely to be ineffective and costly. The time has come to reconsider these recommendations.” | - | +/- | - | NA | NA | NA |
| 18 | Enslin and Kaul | 2020 | US | NA | NA | NA | NA | NA | NA | NA |
| 19 | Esserman et al. | 2014 | US | "Evidence is in short supply to show clinical benefit from endoscopic screening for Barrett’s oesophagus, yet this practice continues." | -/- | - | - | NA | NA | NA |
| 20 | Falk | 2002 | US | NA | NA | NA | NA | NA | NA | NA |
| 21 | Falk | 2019 | US | "Where does this all leave us? High-quality screening for Barrett’s esophagus should currently be offered to men with multiple risk factors. This should be once in a lifetime and the normal appearing Z line should not be biopsied." | ++ | ++ | ++ | NA | NA | NA |
| 22 | Fitzgerald | 2005 | UK | "Detecting high-risk groups, through molecular profiling for example, may be one way forward in order that interventions can be highly targeted. Alternatively, mass screening and chemoprevention strategies may have a greater impact on reducing population mortality. More research is needed in this important area." | + | + | + | NA | NA | NA |
| 23 | Frei et al. | 2021 | NL | “Although the presented EsophaCap and also the Cytosponge appear to be promising techniques that could be ready for prime time in screening for BE, the other necessary requirements to establish a successful BE screening program are still lacking. Only if the screening population can be better defined, target group participation raised, and cost effectiveness of BE surveillance improved will BE screening, in our opinion, be ready for implementation.” | + | +/- | +/- | NA | NA | NA |
| 24 | Shaheen | 2002 | US | "Given the lack of data in support of any kind of mass screening for adenocarcinoma of the esophagus, including endoscopic screening, the authors are justified in their conclusion that more data are necessary to advocate population-based screening to prevent this cancer." | NA | NA | NA | - | - | - |
| 25 | Gerson | 2011 | US | “How do I apply the data gathered from this study into my clinical practice at Stanford? For both men and women with chronic GERD who have undergone a prior normal endoscopic examination, I reassure them that their risk for subsequent development of BE is very low, but that a subsequent screening examination could be considered after 10 years’ time, particularly for white men with chronic GERD, given the lack of data regarding potential BE development.” | + | + | + | NA | NA | NA |
| 26 | Gopal et al. | 2004 | US | “Nevertheless, we, along with most gastroenterologists, advocate screening and surveillance and this is reflected in the clinical practice and guideline recommendations for Barrett’s esophagus published by the major gastroenterology societies.[14] Furthermore, the frequency of examinations should be conducted in accordance with the ACG guidelines, as stated in section 2. Unless the practice of screening and surveillance has clearly been proven ineffective, there is enough evidence to suggest a potential benefit exists, at a low risk of morbidity and mortality to the patient.” | ++ | + | + | NA | NA | NA |
| 27 | Graham et al. | 2016 | UK | "It [endoscopy] should not, however, be offered routinely to the general population." | NA | NA | NA | -/- | -/- | -/- |
| 28 | Graham and Tan | 2020 | US | "We propose (1) improved approaches to Barrett’s prevention, (2) universal Barrett’s screening by linking Barrett’s screening to colon cancer screening, (3) ablation of all Barrett’s mucosa along with (4) acid-suppressive–antireflux therapy tailored to prevent development of Barrett’s or the recurrence of Barrett’s after ablation therapy." | NA | NA | NA | ++ | ++ | ++ |
| 29 | Ilbawi and Anderson | 2015 | US | NA | NA | NA | NA | NA | NA | NA |
| 30 | Iyer and Chak | 2016 | US | "Economic considerations such as absolute direct and indirect medical costs (compared with conventional endoscopy), and overall cost effectiveness of these minimally invasive approaches for the detection of Barrett’s esophagus also need to be defined before widespread adoption becomes a reality." | + | +/- | +/- | NA | NA | NA |
| 31 | de Jonge et al. | 2014 | Other | "At present, however, screening cannot be recommended as the population at risk is too broadly defined, and current screening techniques are burdensome and costly." | -/- | -/- | -/- | NA | NA | NA |
| 32 | Kamboj et al. | 2021 | US | "Screening for BE should be considered in those with multiple risk factors, including age greater than 50 years, male sex, chronic gastroesophageal reflux disease, white race, central obesity, smoking use, first-degree relative with BE or esophageal adenocarcinoma, and presence of hiatal hernia. Screening is not recommended in the general population." | ++ | + | + | -/- | -/- | -/- |
| 33 | Katzka and Fitzgerald | 2020 | UK | "The current paradigm of screening for EAC based on age and the presence of heartburn and use of endoscopy should be reevaluated. The use of less expensive and easier to use esophageal screening devices more applicable to a larger population of patients at risk for EAC should be considered." | + | + | + | NA | NA | NA |
| 34 | Knox | 2011 | US | "Although there is enthusiasm in some groups for screening patients for Barrett’s esophagus, with the goal of reducing death from esophageal adenocarcinoma, review of the evidence does not show sufficient benefit to recommend such a program." | -/- | - | - | NA | NA | NA |
| 35 | Kolb and Wani | 2021 | US | "Nonendoscopic screening methods such as the Cytosponge offer a potentially paradigm-shifting approach to screening for BE and EAC by increasing the proportion of the US population screened for BE. By incorporating a personalized approach to the selection of individuals for screening using these novel approaches and collaboration with primary care providers, we can finally envision decreasing the morbidity and mortality related to EAC." | + | + | + | NA | NA | NA |
| 36 | Konda and Souza | 2019 | US | NA | NA | NA | NA | NA | NA | NA |
| 37 | Kuipers | 2011 | Other | "The existing guidelines are quite clear in their recommendation to exercise restraint when screening patients for Barrett esophagus. The guidelines are also clear in explaining the uncertainties surrounding screening. At the moment, there is no evidence that screening should be performed more widely than recommended by these excellent guidelines." | +/- | + | +/- | NA | NA | NA |
| 38 | Lambert | 2012 | Other | "For esophageal adenocarcinoma in columnar lined esophagus, the risk is too low to justify opportunistic or organized screening." | -/- | - | - | NA | NA | NA |
| 39 | Lao-Sirieix and Fitzgerald | 2012 | UK | "Advances in diagnostic technologies and minimally invasive treatments mean that the time is ripe to seriously consider screening for this disease." | + | + | + | NA | NA | NA |
| 40 | Lieberman/Sampliner | 2001 | US | "It seems that screening all GERD patients is not practical, but if screening can be targeted to high-risk patients, it may be beneficial." | + | + | + | NA | NA | NA |
| 41 | Malagelada | 2011 | Other | "If we discard generalized screening as too expensive and of uncertain value, surveillance of known Barrett’s would seem the logical next step in the preventive strategy ladder, but there are problems associated with this approach as well." | NA | NA | NA |  | - | - |
| 42 | Mehta and Asch | 2014 | US | NA | NA | NA | NA | NA | NA | NA |
| 43 | Michalak et al. | 2009 | US | "The role of screening and surveillance in BE and their cost-effectiveness remain controversial." | - | +/- | +/- | NA | NA | NA |
| 44 | O’Donovan and Fitzgerald | 2018 | UK | "Barrett’s esophagus fulfills many of the Junger criteria for screening now that endoscopic therapy is widely available and proven to be effective. However, it is a relatively uncommon disease compared to colon and breast cancer for example, for which screening is in routine use, and therefore, patient selection is critical in order for the strategy to be cost-effective and acceptable to patients." | + | + | + | NA | NA | NA |
| 45 | Otaki and Iyer | 2018 | US | "While there are several limitations with both screening and surveillance practices, they will likely remain cornerstones of any strategy to reduce the incidence and mortality from EAC. Strong and consistent evidence shows that EAC when diagnosed early is associated with substantially improved outcomes and that EAC can be prevented when dysplasia (low and high grade) is treated endoscopically. Hence effective strategies to detect BE (the only known precursor of EAC) and associated prevalent and incident dysplasia are imperative. While several challenges in both of these interlinked strategies exist, progress is being made in several directions particularly in the development of minimally invasive techniques to screen for BE (which may make BE risk assessment tools less critical, by rendering widespread screening feasible) and the development of efficient sampling and risk stratification tools, which incorporate assessment of biomarkers in cytology or histology samples.." | + | + | + | NA | NA | NA |
| 46 | Patel and Gyawali | 2019 | US | "The selective use of endoscopic screening when risk factors for BE/EAC are identified (with adequate time spent inspecting the esophagus), the use of endoscopic ablative therapies in dysplastic BE, and prospective outcome analysis can maximize cost-effective clinical outcomes."  “However, screening of the general population and of low-risk groups is clearly not cost-effective and not recommended.” | + | + | + |  | -/- | -/- |
| 47 | Rajendra | 2015 | Other | NA | NA | NA | NA | NA | NA | NA |
| 48 | Reid | 2017 | US | "Esophageal adenocarcinoma (EA) is remarkably similar to gastric adenocarcinoma CIN subtype. Current enthusiasm for endoscopic control of EA has little impact on mortality. Current strategies need to be revisited given emerging evidence that many cancers develop rapidly by punctuated and catastrophic genome evolution." | +/- | +/- | +/- | NA | NA | NA |
| 49 | Rubenstein and Thrift | 2015 | US | NA | NA | NA | NA | NA | NA | NA |
| 50 | Sami and Iyer | 2018 | US | "In summary, in addition to the several exciting advances, challenges to the widespread application of BE screening remain. Nevertheless, this area remains ripe for research, particularly focusing on the comprehensive evaluation of emerging minimally-invasive and novel screening tools as well as deriving and validating BE and EAC risk prediction models." | + | + | + | NA | NA | NA |
| 51 | Shaheen et al. | 2002 | US | "Application of these criteria demonstrate that, though some data exist to support a widescale endoscopic screening program of subjects with refux symptoms, the current evidence in favor of such a practice is weak and does not support its implementation." | -/- | -/- | -/- | NA | NA | NA |
| 52 | Shaheen and Palmer | 2009 | US | "After reviewing the body of evidence that describes the utility of currently practiced endoscopic screening programs for BE, one comes to the disappointing conclusion that screening patients who have chronic GERD to identify BE and thus decrease mortality from ACE is a flawed, costly, and ineffective process." | -/- | -/- | -/- | NA | NA | NA |
| 53 | Shaheen | 2011 | US | NA | NA | NA | NA | NA | NA | NA |
| 54 | Sharma and Sidorenko | 2005 | US | “The role of screening and surveillance in patients with Barrett’s oesophagus remains controversial. There is a clear link between screening and surveillance. Patients detected by screening may be committed for further surveillance programmes and this has to be borne in mind before embarking on large scale screening. Existing data do not show that screening for Barrett’s oesophagus is cost effective or improves mortality from oesophageal adenocarcinoma. A critical component of a Barrett’s targeted screening programme will be to identify a high risk group and an inexpensive screening tool.” | -/- | +/- | - | NA | NA | NA |
| 55 | Smith | 2020 | US | NA | NA | NA | NA | NA | NA | NA |
| 56 | Spechler et al., 3 parts by R. Sampliner, P. Moayyedi and J. Spechler | 2004 | US | NA | NA | NA | NA | NA | NA | NA |
| 57 | Spechler et al. | 2018 | US | "We support the current recommendation of GI societies that screening endoscopy for Barrett’s esophagus should be performed only in a well-defined, high-risk population. We do not recommend the use of any alternative test to screen for Barrett’s esophagus at this time. Some of the alternative tests show great promise for Barrett’s screening and will likely find a place in clinical practice in the near future." | + | + | + | NA | NA | NA |
| 58 | Tan et al., | 2021 | UK | “Significant progress has been made in identifying the at-risk population, as well as in the development of new technologies suitable for screening. Prediction algorithms relying on epidemiologic and demographic data are abundant, and work is now required to determine the target population to maximize the yield of screening in a manner that is cost-effective. Newer minimally invasive sampling devices coupled  with biomarkers for diagnosis of BE are highly promising. In the future, the application of risk stratification biomarkers could further help to identify those at greatest risk to avoid unduly burdening endoscopy services. In the future, blood-based biomarkers and breath testing of organic compounds that are currently in development may pave the way for population-based screening if sufficient accuracy profiles can be  achieved.” | + | + | + | NA | NA | NA |
| 59 | Tan and di Pietro | 2022 | UK | “DO’s:  - Screen patients with multiple risk factors for OAC  - Investigate the family history of patients with GORD as a means to identify those at higher risk for BO or OAC  - Consider the use of alternative screening technologies such as Cytosponge or transnasal endoscopy depending on local availability  DON’Ts:  - Endoscope all patients with gastro-oesophageal reflux symptoms” | +/+ | +/+ | +/+ | NA | NA | NA |
| 60 | Wani and Sharma | 2006 | US | "Currently, there is insufficient evidence to accept the proposition that screening for BO improves mortality from adenocarcinoma or is cost-effective." | -/- | - | - | NA | NA | NA |
| 61 | Vaughan and Fitzgerald | 2015 | US | “At the lowest strata there would be minimal intervention and simple, cost-effective tools, whereas in the higher risk groups clinicians would make use of increasingly precise, but also increasingly invasive and expensive tools. Hence, the careful selection of tests and prevention activities at different stages should be favorable economically, in contrast to the current scenario of enrolling more and more patients into endoscopic surveillance with little regard to their absolute risk, and therefore little population benefit.” | +/- | +/- | +/- | NA | NA | NA |
| 62 | Yusuf and Fitzgerald | 2021 | UK | "A three-tier precision cancer programme whereby risk prediction algorithms and non-endoscopic minimally invasive cell collection devices are used to triage test a wider pool of individuals may improve the detection rate of current screening practices with minimal cost implications." | + | + | + | NA | NA | NA |
| 63 | Zakko et al. | 2017 | US | "Only a prospective randomized controlled trial can really determine the benefit though this may not feasible." | +/- | +/- | +/- | NA | NA | NA |

++, recommending; +, motivation; +/-, neutral position; -, serious doubt; -/-, recommending against.

**References**

1. Boolchand V, Faulx A, Das A, et al. Primary care physician attitudes toward endoscopic screening for GERD symptoms and unsedated esophagoscopy. Gastrointestinal Endoscopy 2006;63:228-233.

2. Chey WD, Inadomi JM, Booher AM, et al. Primary-care physicians' perceptions and practices on the management of GERD: Results of a national survey. Am J Gastroenterol 2005;100:1237-1242.

3. Kolb JM, Chen M, Tavakkoli A, et al. Understanding Compliance, Practice Patterns, and Barriers Among Gastroenterologists and Primary Care Providers Is Crucial for Developing Strategies to Improve Screening for Barrett's Esophagus. Gastroenterology 2022;162:1568-1573.e4.

4. Lin OS, Mannava S, Hwang KL, et al. Reasons for current practices in managing Barrett's esophagus. Dis Esophagus 2002;15:39-45.

5. Rubenstein JH, Saini SD, Kuhn L, et al. Influence of malpractice history on the practice of screening and surveillance for Barrett's esophagus. Am J Gastroenterol 2008;103:842-849.

6. Adams MA, Parikh PD, Miller K, et al. Medical professional liability claims related to esophageal cancer screening. JAMA 2014;312:1348-9.

7. AmaDi C, Gatenby P. Barrett's oesophagus: Current controversies. World J Gastroenterol 2017;23:5051-5067.

8. Atkinson M, Chak A. Screening for Barrett's esophagus. Techniques in Gastrointestinal Endoscopy 2010;12:62-66.

9. Blevins CH, Iyer PG. Who Deserves Endoscopic Screening for Esophageal Neoplasia? Gastroenterol Clin North Am 2017;27:365-378.

10. Bretthauer M, Kalager M. Principles, effectiveness and caveats in screening for cancer. British Journal of Surgery 2013;100:55-65.

11. Bretthauer M, Kalager M, Adami HO. Do's and don'ts in evaluation of endoscopic screening for gastrointestinal cancers. Endoscopy 2016;48:75-80.

12. Cook MB. Optimization and expansion of predictive models for barrett's esophagus and esophageal adenocarcinoma: Could a life-course exposure history be beneficial. Am J Gastroenterol 2013;108:923-925.

13. Cook MB, Thrift AP. Epidemiology of Barrett's Esophagus and Esophageal Adenocarcinoma: Implications for Screening and Surveillance. Gastrointestinal Endoscopy Clinics of North America 2021;31:1-26.

14. Craanen ME, Kuipers EJ. Advantages and disadvantages of population screening for cancer and surveillance of at-risk groups. Best Practice and Research: Clinical Gastroenterology 2001;15:211-226.

15. Crockett SD, Barritt IAS, Shaheen NJ. A 52-Year-Old Man With Heartburn: Should He Undergo Screening for Barrett's Esophagus? Clin Gastroenterol Hepatol 2010;8:565-571.

16. Cross S. Barrett esophagus: a practical challenge for primary care. The Nurse practitioner 2011;36:18-21.

17. Dellon ES, Shaheen NJ. Does screening for Barrett's esophagus and adenocarcinoma of the esophagus prolong survival? J Clin Oncol 2005;23:4478-4482.

18. Desai M, Hamade N, Sharma P. Screening for Barrett's esophagus: challenges in identifying the population at risk. Gastrointestinal Endoscopy 2021;93:420-421.

19. Dulai GS, Guha S, Kahn KL, et al. Screening and surveillance in Barrett's esophagus: Much to be known. Evidence-Based Gastroenterology 2002;3:76-77.

20. Di Pietro M, Fitzgerald RC. Screening and Risk Stratification for Barrett's Esophagus. How to Limit the Clinical Impact of the Increasing Incidence of Esophageal Adenocarcinoma. Gastroenterol Clin North Am 2013;42:155-173.

21. Di Pietro M, Chan D, Fitzgerald RC, et al. Screening for Barrett's esophagus. Gastroenterology 2015;148:912-923.

22. di Pietro M, Canto MI, Fitzgerald RC. Endoscopic Management of Early Adenocarcinoma and Squamous Cell Carcinoma of the Esophagus: Screening, Diagnosis, and Therapy. Gastroenterology 2018;154:421-436.

23. Eisen GM, Lieberman D, Fennerty MB, et al. Screening and surveillance in Barrett's esophagus: a call to action. Clin Gastroenterol Hepatol 2004;2:861-4.

24. Enslin S, Kaul V. Barrett's Esophagus Management in the Elderly: Principles and Best Practice. Current Gastroenterology Reports 2020;22.

25. Esserman LJ, Thompson IM, Reid B, et al. Addressing overdiagnosis and overtreatment in cancer: A prescription for change. The Lancet Oncology 2014;15:e234-e242.

26. Falk GW. Barrett's esophagus. Gastroenterology 2002;122:1569-1591.

27. Falk GW. Screening and Surveillance of Barrett's Esophagus: Where Are We Now and What Does the Future Hold? Am J Gastroenterol 2019;114:64-70.

28. Fitzgerald RC. Genetics and prevention of oesophageal adenocarcinoma. Recent Results Cancer Res 2005:35-46.

29. Frei NF, Bergman J, Pouw RE. Screening for Barrett's esophagus: Ready for prime time or still hard to swallow? Gastrointest Endosc 2021;94:506-508.

30. Gerson LB. Are we ready for gender-based guidelines for Barrett's esophagus screening? Gastroenterology 2011;141:2271-2273.

31. Gopal DV, Reichelderfer M, Gaumnitz EA, et al. Barrett's esophagus: Is screening and surveillance justified? Disease Management and Health Outcomes 2004;12:353-361.

32. Graham D, Lipman G, Sehgal V, et al. Monitoring the premalignant potential of Barrett's oesophagus'. Frontline Gastroenterology 2016;7:316-322.

33. Graham DY, Tan MC. No Barrett's-No Cancer: A Proposed New Paradigm for Prevention of Esophageal Adenocarcinoma. J Clin Gastroenterol 2020;54:136-143.

34. Ilbawi AM, Anderson BO. Cancer in global health: How do prevention and early detection strategies relate? Science Translational Medicine 2015;7.

35. Iyer PG, Chak A. Can endosheath technology open primary care doors to Barrett's esophagus screening by transnasal endoscopy? Endoscopy 2016;48:105-6.

36. de Jonge PJ, van Blankenstein M, Grady WM, et al. Barrett's oesophagus: epidemiology, cancer risk and implications for management. Gut 2014;63:191-202.

37. Kamboj AK, Katzka DA, Iyer PG. Endoscopic Screening for Barrett's Esophagus and Esophageal Adenocarcinoma: Rationale, Candidates, and Challenges. Gastrointest Endosc Clin N Am 2021;31:27-41.

38. Katzka DA, Fitzgerald RC. Time to Challenge Current Strategies for Detection of Barrett's Esophagus and Esophageal Adenocarcinoma. Dig Dis Sci 2020;65:18-21.

39. Knox MA. Should we screen patients for Barrett's esophagus? . American family physician 2011;83:1148, 1150.

40. Kolb JM, Wani S. A Paradigm Shift in Screening for Barrett's Esophagus: The BEST Is Yet to Come. Gastroenterology 2021;160:467-469.

41. Konda VJA, Souza RF. Barrett's Esophagus and Esophageal Carcinoma: Can Biomarkers Guide Clinical Practice? Current Gastroenterology Reports 2019;21.

42. Kuipers EJ. Barrett Esophagus and life expectancy: Implications for screening? Gastroenterology and Hepatology 2011;7:689-691.

43. Lambert R. Endoscopy in screening for digestive cancer. World J Gastrointest Endosc 2012;4:518-25.

44. Lao-Sirieix P, Fitzgerald RC. Screening for oesophageal cancer. Nature Reviews Clinical Oncology 2012;9:278-287.

45. Lieberman DA, Sampliner RE. How far to go? screening and surveillance in Barrett's esophagus. American Journal of Managed Care 2001;7:S19-S26.

46. Malagelada JR. Diseases of the digestive tract: Is prevention possible and feasible? Digestive Diseases 2011;29:255-263.

47. Mehta SJ, Asch DA. How to help gastroenterology patients help themselves: leveraging insights from behavioral economics. Clin Gastroenterol Hepatol 2014;12:711-4.

48. Michalak J, Bansal A, Sharma P. Screening and surveillance of Barrett's esophagus. Current Gastroenterology Reports 2009;11:195-201.

49. O'Donovan M, Fitzgerald RC. Screening for Barrett's Esophagus: Are New High-Volume Methods Feasible? Digestive Diseases and Sciences 2018;63:2105-2114.

50. Otaki F, Iyer PG. Point-Counterpoint: Screening and Surveillance for Barrett's Esophagus, Is It Worthwhile? Dig Dis Sci 2018;63:2081-2093.

51. Patel A, Gyawali CP. Screening for Barrett's Esophagus: Balancing Clinical Value and Cost-effectiveness. J Neurogastroenterol Motil 2019;25:181-188.

52. Rajendra S. Barrett's oesophagus: Can meaningful screening and surveillance guidelines be formulated based on new data and rejigging the old paradigm? Best Practice and Research: Clinical Gastroenterology 2015;29:65-75.

53. Reid BJ. Genomics, Endoscopy, and Control of Gastroesophageal Cancers: A Perspective. Cellular and Molecular Gastroenterology and Hepatology 2017;3:359-366.

54. Rubenstein JH, Thrift AP. Risk factors and populations at risk: Selection of patients for screening for Barrett's oesophagus. Best Practice and Research: Clinical Gastroenterology 2015;29:41-50.

55. Sami SS, Iyer PG. Recent Advances in Screening for Barrett's Esophagus. Curr Treat Options Gastroenterol 2018;16:1-14.

56. Shaheen N, Ransohoff DF. Gastroesophageal reflux, barrett esophagus, and esophageal cancer: scientific review. JAMA 2002;287:1972-81.

57. Shaheen NJ, Sharma P, Overholt BF, et al. Radiofrequency ablation in Barrett's esophagus with dysplasia. N Engl J Med 2009;360:2277-88.

58. Shaheen NJ. Should women with heartburn undergo screening upper endoscopy for prevention of cancer. Am J Gastroenterol 2011;106:261-263.

59. Sharma P, Sidorenko EI. Are screening and surveillance for Barrett's oesophagus really worthwhile? Gut 2005;54:i27-i32.

60. Sharma P, Smith MS. New screening methods for barrett esophagus. Gastroenterology and Hepatology 2020;16:590-593.

61. Spechler SJ. Should patients with GERD be screened once at least for Barrett's epithelium? A balancing view: To screen or not to screen: scoping out the issues. Am J Gastroenterol 2004;99:2295-6.

62. Spechler SJ, Katzka DA, Fitzgerald RC. New Screening Techniques in Barrett's Esophagus: Great Ideas or Great Practice? Gastroenterology 2018;154:1594-1601.

63. Tan WK, Sharma AN, Chak A, et al. Progress in Screening for Barrett's Esophagus: Beyond Standard Upper Endoscopy. Gastrointest Endosc Clin N Am 2021;31:43-58.

64. Tan WK, di Pietro M. Barrett's Oesophagus: Today's Mistake and Tomorrow's Wisdom in Screening and Prevention. Visc Med 2022;38:161-167.

65. Wani S, Sharma P. The rationale for screening and surveillance of Barrett's metaplasia. Best Practice and Research: Clinical Gastroenterology 2006;20:829-842.

66. Vaughan TL, Fitzgerald RC. Precision prevention of oesophageal adenocarcinoma. Nat Rev Gastroenterol Hepatol 2015;12:243-8.

67. Yusuf A, Fitzgerald RC. Screening for Barrett's Oesophagus: Are We Ready for it? Current Treatment Options in Gastroenterology 2021:1-16.

68. Zakko L, Visrodia K, Wang KK, et al. The Effect of Bias on Estimation of Improved Survival after Diagnosis of Barrett's Esophagus. Am J Gastroenterol 2017;112:1265-1266.
